# Hospital-based herpes zoster diagnoses in Denmark: rate, patient characteristics, and all-cause mortality

**DOI:** 10.1186/s12879-016-1369-6

**Published:** 2016-03-01

**Authors:** Sigrun A. J. Schmidt, Johnny Kahlert, Mogens Vestergaard, Henrik C. Schønheyder, Henrik T. Sørensen

**Affiliations:** Department of Clinical Epidemiology, Aarhus University Hospital, Olof Palmes Allé 43-45, DK-8200 Aarhus N, Denmark; Research Unit and Section for General Practice, Department of Public Health, Aarhus University, Aarhus, Denmark; Department of Clinical Microbiology, Aalborg University Hospital, Aalborg, Denmark; Department of Clinical Medicine, Aalborg University, Aalborg, Denmark

**Keywords:** Complications, Epidemiology, Herpes zoster, Hospitalization, Outpatient clinics, Hospital

## Abstract

**Background:**

Herpes zoster (HZ) may result in severe complications requiring hospital treatment, particularly in patients with comorbidity. Nevertheless, data on HZ from nationwide population-based hospital registries are sparse.

**Methods:**

We conducted a cohort study describing first-time hospital-based (inpatient, outpatient, and emergency room) HZ diagnoses in the Danish National Patient Registry, 1994–2012. We computed the diagnosis rate; prevalence of demographic characteristics, comorbidities, and complications; length of hospital stay; and standardized mortality ratios (SMRs) using the Danish population as reference. We classified comorbidity using the Charlson Comorbidity Index (CCI) scoring system and categorized patients in groups of no (score 0), moderate (score 1), severe (score 2), and very severe comorbidity (score ≥3). In addition, we computed the prevalence of certain conditions associated with immune dysregulation (stem cell or bone marrow transplantation, solid organ transplantation, HIV infection, primary immunodeficiency, any cancer, and autoimmune diseases).

**Results:**

The diagnosis rate increased almost exponentially from 6 to 91.9 per 100,000 person-years between age 50 and ≥90 years. The age-standardized rate was stable throughout the study period. The median length of hospital stay was 4 days (interquartile range: 1–8 days) for inpatients with HZ as the main reason for admission. According to the CCI, 44.3 % of patients had no comorbidity, 17.3 % moderate comorbidity, 17.4 % severe comorbidity, and 21.0 % very severe comorbidity. Comorbidities involving immune dysregulation, such as malignant (21 %) and autoimmune diseases (17 %), were particularly prevalent. Thirty percent had neurological, ophthalmic, or other complications. HZ was associated with increased all-cause mortality overall (SMR 1.8, 95 % CI: 1.7–1.8), but not in analyses restricted to patients without comorbidity (SMR 1.0, 95 % CI: 0.9–1.0).

**Conclusions:**

This study provides estimates of the epidemiology of hospital-based (severe) HZ. The diagnosis rate increased substantially with age. Complications and comorbidities were prevalent, likely resulting in increased mortality.

**Electronic supplementary material:**

The online version of this article (doi:10.1186/s12879-016-1369-6) contains supplementary material, which is available to authorized users.

## Background

Herpes zoster (HZ) is a neurocutaneous infection caused by reactivation of the varicella-zoster virus (human herpesvirus 3) from the sensory ganglia when cell-mediated immunity wanes below a critical level [1]. In a review of data from nine European countries, the overall incidence rate in the general population was approximately 100–400 per 100,000 person-years in adults aged 50 years or younger. Thereafter, it increased notably, reaching 1000 per 100,000 person-years after age 80 years [2]. The risk of HZ is increased in patients with cell-mediated immunodeficiency, including that caused by drugs (e.g., corticosteroids or chemotherapy), radiotherapy, viral infections (*i.e.*, human immunodeficiency virus [HIV]), or malignancy.

HZ may have severe acute complications, such as meningo-encephalitis and cutaneous dissemination, in particular among individuals with the aforementioned predisposing conditions [3, 4]. These patients are more likely to be treated in the hospital or in hospital outpatient specialty clinics. When assessing the epidemiology of HZ, it is important also to consider these severe or potentially severe cases diagnosed in a hospital-based setting. Nevertheless, results from nationwide population-based hospital registries in Europe are sparse concerning the diagnosis rate and patient characteristics, including prevalence of complications and comorbidities. In particular, none of the previous population-based studies published data pertaining specifically to diagnoses from hospital outpatient specialty clinics. To address this paucity of data, we conducted a nationwide Danish cohort study describing HZ in a hospital-based setting, including diagnosis rate, patient demographics, severity, comorbidity burden, length of admission, and subsequent all-cause mortality.

## Methods

The Danish National Health Service provides tax-funded universal health care to all residents (population 5.6 million), guaranteeing unfettered access to general practitioners and hospitals [5]. Utilization of health services is recorded in nationwide databases using the unique central personal register (CPR) number assigned to each Danish resident at birth or immigration [5]. The CPR number allowed for unambiguous individual-level linkage of the registries used in the current study.

The Danish National Patient Registry (DNPR) contains data on all admissions to Danish non-psychiatric departments since 1977 and visits to hospital-based outpatient clinics (*i.e.*, ambulatory medical care) and emergency rooms since 1994 [6]. Each DNPR record contains the CPR number of the patient, dates of hospital admission and discharge or start and end of outpatient contact, information on procedures performed, and diagnoses. Each record includes a primary diagnosis, *i.e.*, the condition considered the main reason for the hospital contact. Diagnoses are assigned by the discharging physician at the time of hospital discharge or at the end of an outpatient contact using the *International Classification of Diseases*, 8th revision (ICD-8) until the end of 1993 and 10th revision (ICD-10) thereafter [6]. Surgical procedures are coded by the surgeon according to a Danish classification system (1977 through 1995) and a Danish version of the Nordic Medico-Statistical Committee Classification of Surgical Procedures (from 1996 onwards). Since 2002, the DNPR has provided the basis for reimbursing both public and private hospitals. Reporting of all services provided in the private hospital sector became mandatory in 2003. General practitioners and private practice specialists do not report to the DNPR. Diagnosis codes and definitions used are provided in Additional file 1: Table S1.

We used the DNPR to identify all first-time inpatient, outpatient, and emergency room diagnoses of HZ. To ensure complete information on outpatient and emergency room diagnoses and to avoid potential problems associated with the transition between the ICD-8 and ICD-10 coding systems, we chose a study period from January 1, 1994 to December 31, 2012, when ICD-codes were used, exclusively. The date of discharge or end of outpatient contact was considered the diagnosis date. Based on patients’ first-ever HZ diagnosis recorded in the DNPR, we classified HZ diagnoses according to type of hospital-based contact (inpatient, outpatient clinic, or emergency room) and type of diagnosis (primary or secondary). We also identified individual ICD-10 codes for HZ. However, because of the clinical difficulty of distinguishing between HZ encephalitis and meningitis, we combined ICD-10 codes B02.0 and B02.1 in our analysis. Similarly, we grouped ICD-10 codes according to severity (complicated or uncomplicated) and extent (disseminated or localized), as shown in Additional file 1: Table S1. The difference between groups of complicated and disseminated HZ is attributed to codes for HZ ophthalmicus, which is regarded as a localized but complicated form.

To give an overview of reasons for healthcare contacts among patients with a secondary HZ diagnosis, we categorized the primary diagnosis according to a list of 203 morbidity groups based on the ICD-8 and ICD-10 World Health Organization morbidity tables. This code list has been published previously [7] and is outlined in detail in Additional file 1: Table S1. Additional codes outside this list comprised general and unspecific reasons for hospital contact. We grouped them into four categories (‘Symptoms, signs and abnormal clinical and laboratory findings not otherwise specified’, ‘Injury, poisoning and certain other consequences of external causes’, ‘Observation for or follow-up after treatment for cancer’, and ‘Contact with health services due to solid organ or bone marrow transplantation, and ‘Other factors influencing health status and contact with health services’). The HZ vaccine, which is approved by the European Medicines Agency for use in adults aged 50 years or older, did not become available in Denmark until September 2014, and hence not considered in the present study [8].

To quantify comorbidity, we obtained information on all primary, secondary, and additional diagnoses recorded in the DNPR before or on the same date as the HZ diagnosis. We classified comorbidity using the Charlson Comorbidity Index (CCI), a scoring system that assigns between one and six points to 19 groups of chronic diseases (Additional file 1: Table S1) according to their ability to predict death [9]. We obtained information on diagnoses in each disease group. Based on the total CCI score, we also categorized patients into levels of none (score 0), moderate (score 1), severe (score 2), and very severe (score 3 or more) comorbidity. We also obtained information on diagnosis history of conditions associated with immune dysregulation (*i.e.*, stem cell or bone marrow transplantation, solid organ transplantation, HIV infection, primary immunodeficiency, any cancer, and autoimmune diseases) and thus potentially an increased HZ risk [1]. For the categories HIV infection, any cancer, and autoimmune diseases, there was an overlap with codes included in the CCI.

The Danish Civil Registration System (CRS) provides a record of changes in vital and migration status for the entire population since 1968 [5]. We used the CRS to follow HZ patients from diagnosis until the date of death, emigration, or December 31, 2012.

### Statistical analysis

We computed the annual rate of hospital-based HZ diagnoses throughout the study period, with direct age standardization [10] to the 2000 Danish census. We characterized patients according to age, sex, comorbidities, calendar period of diagnosis (1994–1999, 2000–2005, and 2006–2012), and types of HZ diagnoses. For patients with an inpatient primary HZ diagnosis, we computed the median and total length of hospital stay.

We used the Kaplan-Meier estimator to compute absolute mortality risks. We computed standardized mortality ratios (SMRs) with 95 % confidence intervals (CIs) for HZ patients using indirect standardization [10], with the general Danish population as reference. The expected number of deaths was obtained by multiplying the number of person-years at risk in the HZ cohort with the rate of death in the general population according to 5-year age groups, sex, and calendar year. The SMR was computed as the ratio of the observed to the expected number of deaths. Corresponding 95 % CIs were computed assuming that the observed number of deaths followed a Poisson distribution. We performed analyses overall and in subgroups of demographic and patient characteristics.

Analyses were performed using STATA® (version 12.1, STATA, College Station, TX). The study was approved by the Danish Data Protection Agency (record number 1–16–02–1–08). Danish legislation does not require ethical review board approval or informed consent from subjects in registry-based studies.

## Results

### Hospital diagnosis rate

**Fig. 1:**
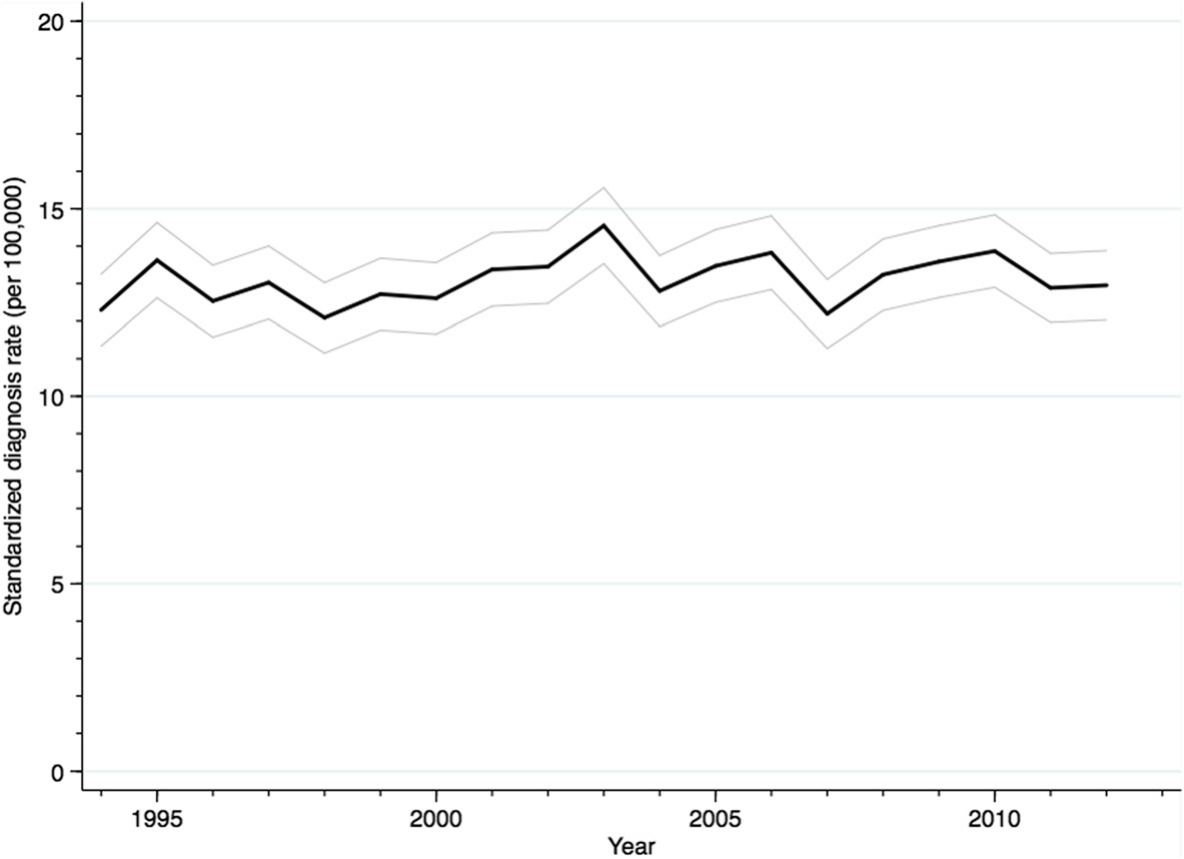
Rates of first-time hospital-based diagnoses of herpes zoster, Denmark, 1994–2012, directly standardized to the age-distribution in the 2000 Danish Census

We identified 13,663 first-time hospital-based diagnoses of HZ in Denmark between 1994 and 2012, corresponding to a standardized HZ rate of 13.1 (95 % CI: 12.9–13.3) per 100,000 person-years (Table 1). The rate was stable throughout the study period, both overall (Fig. 1) and in subgroups defined by age and sex (Additional file 1: Figures S1 and S2).

**Table 1:** Diagnosis rates and median length of stay for hospital-based diagnoses of HZ, Denmark, 1994–2012

Age (years)	Overall			Women			Men			Median length of stay (interquartile range), days^b^
	Number	Person-time	Rate^a^	Number	Person-time	Rate^a^	Number	Person-time	Rate^a^	
0–9	492	12,580,957	3.9	234	6,132,651	3.8	258	6,448,306	4.0	1 (0–3)
10–19	368	12,030,773	3.1	195	5,867,909	3.3	173	6,162,864	2.8	3 (1–6)
20–29	623	13,126,461	4.7	303	6,473,069	4.7	320	6,653,392	4.8	3 (1–6)
30–39	772	14,872,135	5.2	379	7,319,246	5.2	393	7,552,889	5.2	2 (1–5)
40–49	885	14,732,844	6.0	440	7,263,028	6.1	445	7,469,816	6.0	3 (1–6)
50–59	1650	13,516,050	12.2	899	6,726,066	13.4	751	6,789,984	11.1	3 (1–7)
60–69	2357	10,420,187	22.6	1246	5,344,994	23.3	1111	5,075,193	21.9	3 (1–7)
70–79	3094	6,931,652	44.6	1798	3,866,840	46.5	1296	3,064,812	42.3	4 (1–9)
80–89	2859	3,495,850	81.8	1914	2,252,297	85.0	945	1,243,553	76.0	6 (1–11)
≥90	563	617,841	91.1	415	467,360	88.8	148	150,481	98.4	5 (2–10)
Total	13,663	102,324,750	13.4	7823	51,713,460	15.1	5840	50,611,290	11.5	4 (1–8)
Age-standardized^c^			13.1 (12.9–13.3)			13.6 (13.3–13.9)			12.5 (12.2–12.9)	

*Abbreviations: HZ* herpes zoster

^a^Per 100,000 person-years

^b^For inpatient primary diagnoses only

^c^Directly standardized to the age distribution in the 2000 Danish Census using the following weights: 0.13 for 0–9 years, 0.11 for 10–19 years, 0.14 for 20–29 years, 0.15 for 30–39 years, 0.14 for 40–49 years, 0.14 for 50–59 years, 0.09 for 60–69 years, 0.07 for 70–79 years, 0.03 for 80–89 years, and 0.01 for ≥90 years

**Fig. 2:**
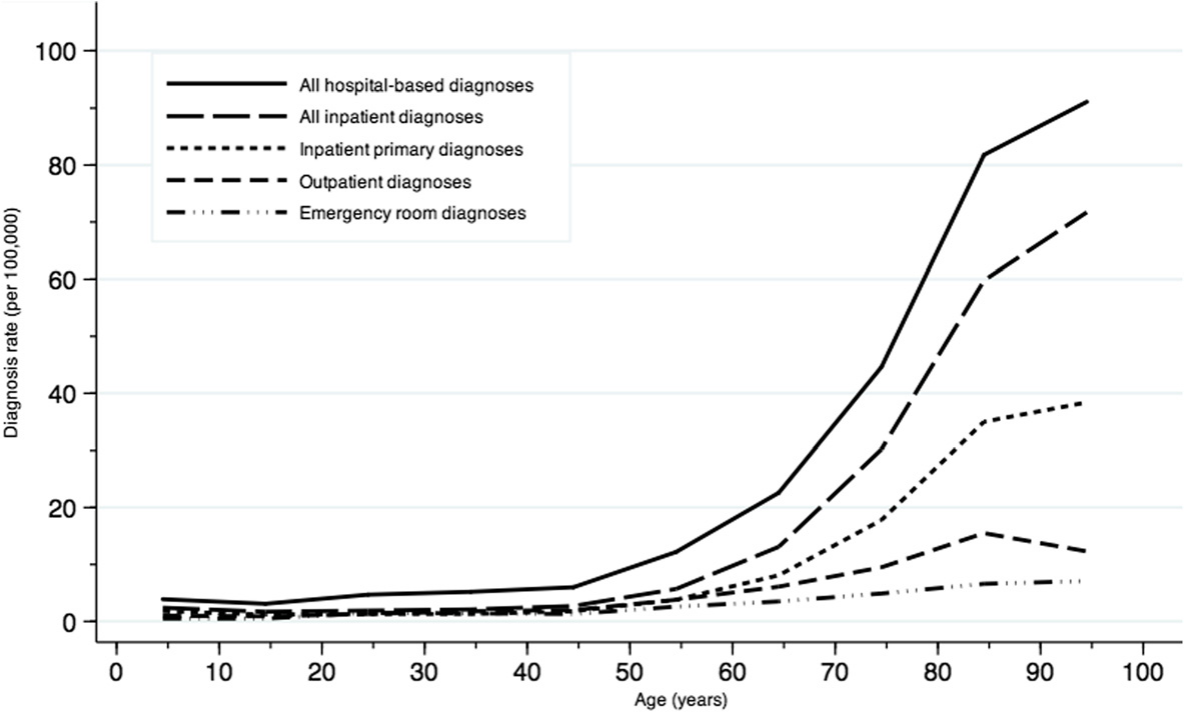
Age-specific rates of hospital-based diagnoses of herpes zoster according to diagnosis types, Denmark, 1994–2012

The HZ rate varied markedly with age. The rate was 3–6 per 100,000 person-years for persons aged below 50 years after which it increased approximately twofold with each decade of age, reaching 91.1 per 100,000 person-years for those aged 90 years or older (Table 1). For all individuals aged 50 years or more (*i.e.*, those potentially eligible for the HZ vaccine), the overall diagnosis rate was 30.1 per 100,000 person-years. The increase in rate with age was observed for all subtypes of HZ diagnoses, but was most pronounced for inpatient diagnoses (Fig. 2).

The standardized diagnosis rate was slightly higher among women than men (13.6 vs. 12.5 per 100,000 person-years). This difference was primarily driven by a difference in rates for persons aged 50–89 years (Table 1).

### Type of diagnosis, complications, and length of stay

Sixty percent of patients were diagnosed with HZ during a hospital admission, 25 % in an outpatient clinic, and 15 % in the emergency room (Table 2). The majority of diagnoses were primary (72 %). Reasons for hospital contact among patients with secondary HZ diagnoses are listed in Additional file 1: Table S2; the most frequent were the unspecific codes ‘other factors influencing health status and contact with health services’ (8.1 %) and ‘symptoms, signs, and abnormal clinical and laboratory findings’ (8.0 %), followed by ‘pneumonia’ (5.9 %), and ‘bronchitis, emphysema and other obstructive pulmonary diseases’ (4.8 %). In total, cancer was the primary diagnosis in 9.5 % of patients with a secondary HZ diagnosis.

**Table 2:** No. (%) of types of first-time hospital-based diagnoses of HZ, Denmark, 1994–2012

	Calendar period						Total	
	1994–1999		2000–2005		2006–2012			
Type of hospital contact								
Emergency room	765	(19)	655	(15)	610	(12)	2030	(15)
Outpatient clinic	911	(23)	1035	(24)	1459	(28)	3405	(25)
Inpatient	2323	(58)	2661	(61)	3244	(61)	8228	(60)
Type of diagnosis								
Primary diagnosis	3040	(76)	3036	(70)	3759	(71)	9835	(72)
Secondary diagnosis	959	(24)	1315	(30)	1554	(29)	3828	(28)
Severity of HZ								
Uncomplicated	2873	(72)	3178	(73)	3580	(67)	9631	(70)
Complicated	1126	(28)	1173	(27)	1733	(33)	4032	(30)
Extent of HZ								
Localized	3390	(85)	3708	(85)	4235	(80)	11,333	(83)
Disseminated	609	(15)	643	(15)	1078	(20)	2330	(17)
Diagnosis code (ICD-10 code)								
HZ meningo-encephalitis (B02.0–1)	62	(1.6)	79	(1.8)	196	(3.7)	337	(2.5)
HZ encephalitis (B02.0)	54	(1.4)	63	(1.4)	146	(2.7)	263	(1.9)
HZ meningitis (B02.1)	8	(0.20)	16	(0.37)	50	(0.94)	74	(0.54)
HZ with other nervous system involvement (B02.2)	323	(8.1)	320	(7.4)	418	(7.9)	1061	(7.8)
HZ ophthalmicus (B02.3)	517	(13)	530	(12)	655	(12)	1702	(12)
Disseminated HZ (B02.7)	74	(1.9)	90	(2.1)	109	(2.1)	273	(2.0)
HZ with other complication (B02.8)	150	(3.8)	154	(3.5)	355	(6.7)	659	(4.8)
HZ without complication (B02.9)	2873	(72)	3178	(73)	3580	(67)	9631	(71)
Total	3999	(29)	4351	(32)	5313	(39)	13,663	(100)

*Abbreviations: HZ* herpes zoster, *ICD-10* International Classification of Diseases, 10th revision

Complicated HZ occurred among 30 % of patients, the most frequent being HZ ophthalmicus followed by nervous system involvement (Table 2). There was a slight increase in the incidence and proportion of patients with ‘HZ encephalitis’, ‘HZ meningitis’ and ‘HZ with other complication’ during the study period. Almost half of the outpatient HZ diagnoses were complicated (28 % were HZ ophthalmicus), compared with one-fourth of inpatient and emergency room diagnoses (Additional file 1: Table S3).

The median length of stay for inpatients with a primary HZ diagnosis was 4 days (interquartile range 1–8 days, mean 6.2 days), increasing with age (Table 1). On average, patients with primary inpatient HZ diagnoses had an annual total of 1634 inpatient days, of which 88 % were in patients aged 50 years or more. The longest stays were observed in patients with complicated HZ, especially HZ encephalitis (median 10 days, interquartile range 6–18 days, mean 15.5 days).

### Comorbidity

A large proportion of persons with HZ had comorbidity. As measured by the CCI, 44 % had no comorbidity, 17 % had moderate comorbidity, 17 % had severe comorbidity, and 21 % had very severe comorbidity (Table 3). The most prevalent diagnoses were autoimmune diseases (17 %), chronic pulmonary disease (14 %), non-metastatic solid tumor (13 %), cerebrovascular disease (11 %), congestive heart failure (7.7 %), connective tissue disease (7.2 %), myocardial infarction (6.7 %), diabetes without end-organ damage (6.7 %), ulcer disease (6.2 %), and lymphoma (6.1 %). In total, 21 % had an active or previous cancer. Diseases possibly associated with immune dysregulation were recorded in 36 % of HZ patients. The prevalence of HIV infection was 1.5 %. Median age and comorbidity burden were highest in patients with inpatient and secondary HZ diagnoses (Additional file 1: Table S3).

**Table 3:** Characteristics of 13,663 patients with a first-time hospital-based diagnosis of HZ, Denmark, 1994–2012

Characteristic	No.	Percent
Age group (years)^a^		
0–9	492	(3.6)
10–19	368	(2.7)
20–29	623	(4.6)
30–39	772	(5.7)
40–49	885	(6.5)
50–59	1650	(12)
60–69	2357	(17)
70–79	3094	(23)
80–89	2859	(21)
≥ 90	563	(4.1)
Sex		
Women	7823	(57)
Men	5840	(43)
Any of the comorbidities identified	8067	(59)
Charlson Comorbidity Index level		
None	6058	(44)
Moderate	2364	(17)
Severe	2374	(17)
Very severe	2867	(21)
Comorbidities in the Charlson Comorbidity Index		
Myocardial infarction	921	(6.7)
Congestive heart failure	1050	(7.7)
Peripheral vascular disease	803	(5.9)
Cerebrovascular disease	1446	(11)
Dementia	256	(1.9)
Chronic pulmonary disease	1911	(14)
Connective tissue disease	983	(7.2)
Ulcer disease	848	(6.2)
Mild liver disease	171	(1.3)
Diabetes without end-organ damage	918	(6.7)
Hemiplegia	57	(0.42)
Moderate to severe renal disease	643	(4.7)
Diabetes with end-organ damage	419	(3.1)
Non-metastatic solid tumor	1755	(13)
Leukemia	489	(3.6)
Lymphoma	834	(6.1)
Moderate to severe liver disease	62	(0.45)
Metastatic solid tumor	331	(2.4)
Acquired immune deficiency syndrome	153	(1.1)
Comorbidities associated with immune dysregulation	4942	(36)
Stem cell or bone marrow transplant	273	(2.0)
Solid organ transplant	152	(1.1)
Human immunodeficiency virus infection	210	(1.5)
Primary immunodeficiency	61	(0.45)
Any cancer	2821	(21)
Any autoimmune disease	2293	(17)
Hematologic	73	(0.53)
Endocrine	614	(4.5)
Central nervous system	67	(0.49)
Gastrointestinal	274	(2.0)
Skin	315	(2.3)
Connective tissue disease	1080	(7.9)
Pulmonary	31	(0.23)
Ocular	94	(0.69)

*Abbreviations: HZ* herpes zoster

^a^Overall, the age range was 0 to 103 years. Median age was 68 years: 71 years in women and 65 years in men

### Mortality

The 30-day mortality for persons with HZ was 3 %, corresponding to 7 % (434/6059) of all deaths in the cohort in total. We observed higher mortality in patients with HZ compared with the general population (Table 4). The overall SMR was 1.8 (95 % CI: 1.7–1.8): 1.6 (95 % CI: 1.6–1.7) in women and 2.0 (95 % CI: 1.9–2.1) in men. The highest SMRs were found in young patients. However, after stratifying by comorbidity level, the SMR was not increased in patients without comorbidity (SMR 1.0; 95 % CI: 0.9–1.0). SMRs according to subtypes of HZ diagnoses are shown in Additional file 1: Table S4.

**Table 4:** SMRs among patients with a first-time hospital-based diagnosis of HZ, Denmark, 1994–2012

	Observed	Expected	SMR (95 % CI)^a^
Overall	6059	3414	1.8 (1.7–1.8)
Age group (years)			
0–9	12	1.1	10.7 (6.1–18.9)
10–19	18	1.1	16.4 (10.4–26.1)
20–29	34	4.0	8.5 (6.1–12.0)
30–39	66	11	5.8 (4.6–7.4)
40–49	143	30	4.8 (4.1–5.7)
50–59	451	124	3.6 (3.3–4.0)
60–69	916	350	2.6 (2.5–2.8)
70–79	1874	988	1.9 (1.8–2.0)
80–89	2080	1507	1.4 (1.3–1.4)
≥ 90	465	397	1.2 (1.1–1.3)
Sex			
Women	3530	2166	1.6 (1.6–1.7)
Men	2529	1248	2.0 (1.9–2.1)
Charlson Comorbidity Index level			
None	1663	1651	1.0 (1.0–1.1)
Moderate	1229	784	1.6 (1.5–1.7)
Severe	1346	506	2.7 (2.5–2.8)
Very severe	1821	473	3.8 (3.7–4.0)
Comorbidity associated with immune dysregulation			
No	3378	2513	1.3 (1.3–1.4)
Yes	2681	901	3.0 (2.9–3.1)
Any of the identified comorbidities			
No	1532	1555	1.0 (0.9–1.0)
Yes	4527	1858	2.4 (2.4–2.5)

*Abbreviations: CI* confidence interval, *HZ* herpes zoster, *SMR* Standardized mortality ratios

^a^Computed using indirect standardization with the Danish general population as reference. Due to rounding the estimates and upper and lower confidence bounds may appear the same

## Discussion

In this nationwide Danish study, the rate of hospital-based HZ diagnoses was stable at around 13 per 100,000 person-years over a study period of 19-years. The rate was 3–6 per 100,000 person-years for persons aged below 50 years, increasing by approximately twofold with each consecutive decade of age. Thirty percent of patients had complicated HZ and comorbidity was prevalent, particularly diseases involving immune dysregulation.

The age-specific hospitalization rates from the present study and previous European studies are shown in Additional file 1: Figure S3. While the overall and age-specific inpatient hospital diagnosis rates in Denmark accord with previous studies [11–24], however there is considerable variation across countries. This variation may result from differences in hospital admission practices or health-seeking behavior, as the overall incidence rate in the general population varies only slightly across Europe [2]. Nevertheless, the nearly exponential increase in the rate of hospital-based HZ diagnoses after age 50 years [11–18, 20–23] and the female predominance [12, 13, 16, 18, 19, 21] in our study are comparable with results from the previous studies. However, none of these studies presented data specifically on HZ diagnosed in hospital-based outpatient clinics, which accounted for one-fourth of patients in our study.

We observed a shorter length of hospital stay (mean 6.2 days) compared to previous studies (mean 7.5 to 11.2 days) [12, 13, 17–19, 22, 25, 26]. Our result was closest to that reported in a Swedish study [18], which also had a similar diagnosis rate and proportion of complicated infections. The shorter length of stay may be explained by hospitalization of milder cases of HZ in Denmark and Sweden, as the proportion of complicated infections (mainly ocular and neurological) was approximately twice as high in Southern Europe [12, 14, 19, 20, 25, 26]. Studies from Southern Europe also reported a higher prevalence of predisposing conditions, such as HIV [12, 14, 16, 20], organ transplantation [12, 16], diabetes [16], and ‘blood dyscrasias and some immune deficiencies’ [16] in HZ patients. Frequencies of malignancy [14, 16, 20] and connective tissue disease [16] were comparable to the results in our study. Finally, the length of stay for patients with neurological complications in our data accords with Italian and French data [19, 25, 26], further suggesting that differences in admission practices play a role. The slight temporal increase in the diagnosis rate of HZ encephalitis and meningitis in our study may result from increased use of polymerase chain reaction technology for detecting varicella-zoster virus in cerebrospinal fluid [27].

Chronic pulmonary disease, connective tissue disease, cancer, HIV, and solid organ transplantation were relatively more frequent in our study compared with other selected Danish patient populations [28–31]. This finding is consistent with the increased risk of HZ in patients with immune dysregulation due to either disease pathophysiology or treatment (e.g., with glucocorticoids) [1]. Apart from posing an additional burden to these patients, HZ could complicate their treatment, for instance by delaying chemotherapy for cancer. We lacked data on HZ treated in general practice and had no comparison cohort, which prevented us from examining whether HZ was associated with increased mortality in patients with certain comorbidities. Although we found increased mortality among patients with comorbidity, we obtained this result using standardization to the entire general population, including those without comorbidity. Thus, the increased SMRs may potentially reflect excess mortality from the comorbid condition rather than from an interaction between HZ and the comorbid condition. The difference in SMRs by age could also be explained by use of the general population as reference. A high prevalence of the exposure (HZ) in the general population would bias the SMR towards the null [32]. Because the prevalence of HZ increases with age, the risk of this bias also may increase with age, potentially explaining our finding of lower SMRs in the oldest age groups.

We used a comprehensive nationwide database to study hospital-based HZ diagnoses within a universal healthcare system, which limits selective inclusion of specific hospitals, insurance groups, or age groups. Nonetheless, our study has some limitations. Since 2002, reimbursements to public and private hospitals have been administered using the Diagnosis Related Group (DRG) system in the DNPR [6], which may have affected physicians’ choice of primary and secondary diagnosis codes. As well, the completeness and validity of HZ diagnoses in the DNPR is unknown. Still, other studies have found high diagnostic accuracy with positive predictive values ranging from 72 to 100 % [33–37]. Although there is a specific code for specifying consultation with post-herpetic neuralgia (ICD-10 G53.0), it is possible that some codes, particularly ‘other nervous system involvement’, were assigned alone to patients who had this chronic complication rather than acute HZ. However, we consider such misclassification of minor importance because these patients experienced recent varicella-zoster virus reactivation.

Our comorbidity assessment has some limitations. The positive predictive value for diagnoses included in the CCI are known to vary depending on the disease, department of origin, and reference standard [6]. Moreover, incompleteness is expected for conditions that are treated primarily in general practice, e.g., diabetes. Another concern is underestimation early in the study period due to left-censoring of outpatient and emergency room diagnoses before 1994. Similarly, changes in coding when new medical treatments are introduced may explain the lack of HZ patients with bone marrow transplantations early in the study period.

The majority of patients with HZ in Denmark are treated in general practices [38], which are not subject to systematic monitoring in national registries. Thus, analysis of temporal trends in our study may not reflect true changes in disease incidence in the population. Administrative factors (e.g., changing admission practices) are also important in determining whether patients receive a hospital-based diagnosis. As well, the lack of primary care data may have caused underestimation of the SMRs [32].

## Conclusions

This nationwide Danish study provided pre-vaccination estimates of the epidemiology of hospital-based HZ. Because hospital-based treatment is likely linked to presence of complications or underlying diseases, our study likely comprises the most severe cases of HZ. Indeed, 30 % of patients had complicated HZ and diseases associated with increased risk of severe HZ (e.g., hematological cancer [3, 4]) were prevalent. These results stress the importance of considering hospital-based diagnoses in studies assessing the overall burden of HZ in the population.

## Availability of data and materials

No additional data are available. According to the Danish Act on Processing of Personal Data, private and public institutions may obtain the health data used in the current study, after obtaining the necessary project-specific approvals.

## Additional file

Additional file 1:
**Supplementary figures and tables.** (PDF 886 kb)

## Abbreviations

CI: confidence interval
CPR: central personal register
DNPR: Danish National Patient Registry
ICD: International Classification of Diseases
HIV: human immunodeficiency virus
HZ: herpes zoster
SMR: standardized mortality ratio
