# Supplementary material for: Hospital-based herpes zoster diagnoses in Denmark: rate, patient characteristics, and all-cause mortality
Source: BMC Infect Dis. 2016 Mar 1;16:99. doi: 10.1186/s12879-016-1369-6 (PMC4773995; doi:10.1186/s12879-016-1369-6)
Supplement: Additional file 1: — Supplementary figures and tables. (PDF 886 kb) [file 12879_2016_1369_MOESM1_ESM.pdf]

ADDITIONAL FILE

- Table S1. Registry codes used in the study
- Table S2. Reasons for hospital contact among patients with a secondary herpes zoster diagnosis, Denmark, 1994–2012
- Table S3. Characteristics of 13,663 patients with a first-time hospital-based diagnosis of HZ, Denmark, 1994–2012, by diagnosis types
- Table S4. SMRs among patients with a first-time hospital-based diagnosis of HZ, Denmark, 1994–2012, by diagnosis types
- Figure S1. Rate of first-time hospital-based diagnoses of herpes zoster among men and women, Denmark, 1994–2012, directly standardized to the age-distribution in the 2000 Danish Census
- Figure S2. Age-specific rates of first-time hospital-based diagnoses of herpes zoster, Denmark, 1994–2012
- Figure S3. Age-specific diagnosis rates of herpes zoster reported for European countries

Table S1. Registry codes used in the study

**Herpes zoster, all diagnoses**	ICD-8: 053.00–053.9; ICD-10: B02.0–B02.9
Complicated	Herpes zoster encephalitis: ICD-10: B02.0<br>Herpes zoster meningitis: ICD-10: B02.1<br>Herpes zoster with other complication of nervous system: ICD-10: B02.2<br>Herpes zoster in eye: ICD-10: B02.3<br>Herpes zoster disseminated: ICD-10: B02.7<br>Herpes zoster with other complication: ICD-10: B02.8
Uncomplicated	Herpes zoster without complication: ICD-10: B02.9
Localized	Herpes zoster in eye: ICD-10: B02.3<br>Herpes zoster without complication: ICD-10: B02.9
Disseminated	Herpes zoster encephalitis: ICD-10: B02.0<br>Herpes zoster meningitis: ICD-10: B02.1<br>Herpes zoster with other complication of nervous system: ICD-10: B02.2<br>Herpes zoster disseminated: ICD-10: B02.7<br>Herpes zoster with other complication: ICD-10: B02.8
**Stem cell or bone marrow tranplantation**	
Autologous	Treatment codes: BOQE1–BOQE2, BOQF0; ICD-10: Z948C2
Allogeneic	Treatment codes: BOQE3–BOQE7, BOQF1–2, BWHA32; ICD-10: Z948C1
Autologous or allogeneic stem cell	If any of the following<br>(1) Treatment codes: BOQE–BOQF if not specified further;<br>(2) Treatment codes: BWHA31;<br>(3) ICD-10: Z948C if not specified further as Z948C1 or Z948C2;<br>(4) ICD-10: Z948 (if not specified further as Z948C, Z948C1, or Z948C2) if coded together with one of the following A–diagnoses C770, C81–C96, D45–D47, D50–D85, D87–D89, T86.0, T86.0A, or T88.8N.
**Solid organ transplantation**	Danish Classification of Surgical Procedures: 322.09, 322.29, 322.50, 356.09, 472.70, 472.79, 488.40, 488.49, 574.80, 574.90; NOMESCO Classification of Surgical Procedures: FQ, GDG, JLE, JJC, KAS
**Human immunodeficiency virus infection**	ICD-8: 079.83, Y40.49, Y41.49; ICD-10: B20–B24, F02.4
**Primary immunodeficiency**	ICD-10: D80–D84, D89
**Autoimmune diseases**	
Hematological system	
Autoimmune hemolytic anemia	ICD-8: 283.90; ICD-10: D59.0, D59.1
Idiopathic thrombocytopenic purpura	ICD-8: 287.10; ICD-10: D69.3

Endocrine system	
Graves' disease	ICD-8: 242.00, 242.01, 242.08, 242.09; ICD-10: E05.0
Autoimmune thyroditis	ICD-8: 244.01, 245.03; ICD-10: E06.3
Addison´s disease	ICD-8: 255.10; ICD-10: E27.1
Diabetes type I	ICD-8: 249; ICD-10: E10
Central nervous/ neuromuscular system	
Multiple sclerosis	ICD-8: 340; ICD-10: G35
Myasthenia gravis	ICD-8: 733.09; ICD-10: G70.0
Gastrointestinal/ hepatobiliary system	
Pernicious anemia	ICD-8: 281.00, 281.01, 281.08, 281.09; ICD-10: D51.0
Coelic disease	ICD-8: 269.00; ICD-10: K90.0
Crohn´s disease	ICD-8: 563.01, 563.02, 563.09; ICD-10: K50, M07.4
Ulcerative colitis	ICD-8: 563.19, 569.04; ICD-10: K51, M07.5
Primary biliary cirrhosis	ICD-8: 571.90; ICD-10: K74.3
Skin	
Atopic dermatitis	ICD-8: 691.00; ICD-10: L20
Pemphigus/ pemphigoid	ICD-8: 694.00–694.03, 694.05; ICD-10: L10.0, L10.1, L10.2, L10.4, L12.0
Dermatitis herpetiformis	ICD-8: 693.08, 693.09; ICD-10: L13.0
Psoriasis	ICD-8: 696.09, 696.10, 696.19; ICD-10: L40, M07.0–M07.3
Vitiligo	ICD-8: 709.01; ICD-10: L80
Connective tissue diseases	
Rheumatoid arthritis	ICD-8: 712.19, 712.29, 712.39, 712.59; ICD-10: M05, M06, G73.7D, I32.8A, I39.8E, I41.8A, I52.8A
Juvenile rheumatoid arthritis	ICD-8: 712.09; ICD-10: M08
Ankylosing spondylitis	ICD-8: 712.49; ICD-10: M45, H221B
Polymyositis/ dermatomyositis	ICD-8: 716.09, 716.19; ICD-10: M33
Systemic– and subacute cutaneous lupus erythematosus	ICD-8: 734.19; ICD-10: M32, G05.8A, G73.7C, I32.8B, I39.8C, L93.1, L93.2, N08.5A, N16.4B
Systemic scleroderma	ICD-8: 734.00–734.09; ICD-10: M34.0–34.9
Mixed connective tissue disease	ICD-8: 734.91; ICD-10: M35.1
Sjögren´s syndrome	ICD-8: 734.90; ICD-10: M35.0, G73.7A, N16.4A
Sarcoidosis	ICD-8: 135.99; ICD-10: D86, G53.2, H22.1A, I41.8B, K77.8B, M63.3
Vasculitis syndromes including polymyalgia rheumatica	ICD-8: 287.09, 446.09–446.99; ICD-10: D69.0B, I77.6, L95, M30–M31, M35.3, M35.6, M79.3, N08.5B–N08.5E

Pulmonary system	
Idiopathic fibrosing alveolitis (pulmonary fibrosis)	ICD-8: 517.01; ICD-10: J841A, J841B, J841C
Ocular diseases	
Iridocyclitis	ICD-8: 364; ICD-10: H200, H201
Any autoimmune disease	If any of the codes listed above
**Charlson Comorbidity Index**	
Score 1	
Myocardial infarction	ICD-8: 410; ICD-10: I21, I22, I23
Congestive heart failure	ICD-8: 427.09, 427.10, 427.11, 427.19, 428.99, 782.49; ICD-10: I50, I11.0, I13.0, I13.2
Peripheral vascular disease	ICD-8: 440, 441, 442, 443, 444, 445; ICD-10: I70, I71, I72, I73, I74, I77
Cerebrovascular disease	ICD-8: 430–438; ICD-10: I60–I69, G45, G46
Dementia	ICD-8: 290.09–290.19, 293.09; ICD-10: F00–F03, F05.1, G30
Chronic pulmonary disease	ICD-8: 490–493, 515–518, ICD-10: J40–J47, J60–J67, J68.4, J70.1, J70.3, J84.1, J92.0, J96.1, J98.2, J98.3
Connective tissue disease	ICD-8: 712, 716, 734, 446, 135.99; ICD-10: M05, M06, M08, M09, M30, M31, M32, M33, M34, M35, M36, D86
Ulcer disease	ICD-8: 530.91, 530.98, 531–534; ICD-10: K22.1, K25–K28
Mild liver disease	ICD-8: 571, 573.01, 573.04; ICD-10: B18, K70.0–K70.3, K70.9, K71, K73, K74, K76.0
Diabetes type 1 and 2	ICD-8: 249.00, 249.06, 249.07, 249.09, 250.00, 250.06, 250.07, 250.09; ICD-10: E10.0, E10.1, E10.9, E11.0, E11.1, E11.9
Score 2	
Hemiplegia	ICD-8: 344; ICD-10: G81, G82
Moderate to severe renal disease	ICD-8: 403, 404, 580–583, 584, 590.09, 593.19, 753.10–753.19, 792; ICD-10: 12, I13, N00–N05, N07, N11, N14, N17–N19, Q61
Diabetes with end-organ damage	ICD-8: 249.01–249.05, 249.08, 250.01–250.05, 250.08; ICD-10: E10.2–E10.8, E11.2–E11.8
Any tumor	ICD-8: 140–194; ICD-10: C00–C75
Leukemia	ICD-8: 204–207; ICD-10: C91–C95
Lymphoma	ICD-8: 200–203, 275.59; ICD-10: C81–C85, C88, C90, C96
Score 3	
Moderate to severe liver disease	ICD-8: 070.00, 070.02, 070.04, 070.06, 070.08, 573.00, 456.00–456.09; ICD-10: B15.0, B16.0, B16.2, B19.0, K70.4, K72, K76.6, I85
Score 6	

Metastatic solid tumor	ICD-8: 195–198, 199; ICD-10: C76–C80
Acquired immune deficiency syndrome	ICD-8: 079.83; ICD-10: B21–B24
**Broad grouping of primary diagnoses in patients with secondary herpes zoster diagnosis**[a]	
Cholera	ICD-8: 0.0–0.9<br>ICD-10: A00.0–A00.9
Typhoid and paratyphoid fevers	ICD-8: 1.0–1.9<br>ICD-10: A01.0–A01.9
Other intestinal infectious diseases	ICD-8: 5.0–5.9<br>ICD–8: 7.0-7.9<br>ICD-10: A02.0–A02.9<br>ICD-10: A04.0–A05.9<br>ICD-10: A07.0–A08.9
Shigellosis/Bacillary dysentery	ICD-8: 4.0–4.9<br>ICD-10: A03.0–A03.9
Amoebiasis	ICD-8: 6.0–6.9<br>ICD-10: A06.0–A06.9
Diarrhea and gastro-enteritis of presumed infectious origin	ICD-8: 8.0–9.9<br>ICD-10: A09.0–A09.9
Respiratory tuberculosis	ICD-8: 10.0–12.3<br>ICD-10: A15.0–A16.9
Other tuberculosis	ICD-8: 13.0–19.9<br>ICD-10: A17.0–A19.9<br>ICD-10: B90.0–B90.9
Plague	ICD-8: 20.0–20.9<br>ICD-10: A20.0–A20.9
Other bacterial diseases	ICD-8:21.0–22.9<br>ICD-8: 24.0–27.9<br>ICD-8: 31.0–31.9<br>ICD-8: 34.0–34.1<br>ICD-8: 35.0–35.9<br>ICD-8: 30.0–39.9<br>ICD-10: A21.0–A22.9<br>ICD-10: A24.0–A28.9<br>ICD-10: A31.0–A32.9<br>ICD-10: A38.0–A38.9<br>ICD-10: A42.0–A49.9<br>ICD-10: B96.0–B96.9
Brucellosis	ICD-8: 23.0–23.9<br>ICD-10: A23.0–A23.9
Leprosy	ICD-8:30.0–30.9<br>ICD-10: A30.0–A30.9<br>ICD-10: B92.0–B92.9
Tetanus	ICD-8: 37.0–37.9<br>ICD-10: A33.0–A33.9<br>ICD-10: A34.0–A35.9

Diphtheria	ICD-8: 32.0–32.9<br>ICD-10: A36.0–A36.9
Whooping cough	ICD-8: 33.0–33.9<br>ICD-10: A37.0–A37.9
Meningococcal infection	ICD-8: 36.0–36.9<br>ICD-10: A39.0–A39.9
Septicemia	ICD-8: 38.0–38.9<br>ICD-10: A40.0–A41.9
Early syphilis	ICD-8: 91.0–91.9<br>ICD-10: A51.0–A51.9
Other syphilis	ICD-8: 90.0–90.9<br>ICD-8: 92.0–97.9<br>ICD-10: A50.0–A50.9<br>ICD-10: A52.0–A53.9
Gonococcal infection	ICD-8: 98.0–98.9<br>ICD-10: A54.0–A54.9
Other infectious and parasitic diseases	ICD-8: 130.0–136.9<br>ICD-8: 89.0–89.9<br>ICD-8: 99.0–117.9<br>ICD-10: A55.0–A67.9<br>ICD-10: A69.0–A70.9<br>ICD-10: A74.0–A74.9<br>ICD-10: A77.0–A79.9<br>ICD-10: B35.0–B49.9<br>ICD-10: B58.0–B64.9<br>ICD-10: B85.0–B89.9<br>ICD-10: B94.0–B94.9<br>ICD-10: B99.0–B99.9
Relapsing fevers	ICD-8: 88.0–88.9<br>ICD-10: A68.0–A68.9
Other viral diseases	ICD-8: 45.0–46.9<br>ICD-8: 50.0–54.9<br>ICD-8: 57.0–57.9<br>ICD-8: 61.0–61.9<br>ICD-8: 66.0–66.9<br>ICD-8: 68.0–68.9<br>ICD-8: 73.0–79.9<br>ICD-10: A71.0–A71.9<br>ICD-10: A81.0–A81.9<br>ICD-10: A87.0–A89.9<br>ICD-10: B00.0–B04.9<br>ICD-10: B07.0–B09.9<br>ICD-10: B20.0–B25.9<br>ICD-10: B27.0–B34.9<br>ICD-10: B97.0–B97.9
Typhus and other rickettsioses	ICD-8: 80.0–83.9<br>ICD-10: A75.0–A75.9
Acute poliomyelitis	ICD-8: 40.0–44.9<br>ICD-10: A80.0–A80.9

	ICD-10: B91.0–B91.9
Rabies	ICD-8: 71.0–71.9<br>ICD-10: A82.0–A82.9
Viral encephalitis	ICD-8: 62.0–65.9<br>ICD-10: A83.0–A86.9
Other arthropod-borne viral fevers and viral hemorrhagic fevers	ICD-8: 67.0–67.9<br>ICD-10: A90.0–A94.9<br>ICD-10: A96.0–A99.9
Yellow fever	ICD-8: 60.0–60.9<br>ICD-10: A95.0–A95.9
Measles	ICD-8: 55.0–55.9<br>ICD-10: B05.0–B05.9
Rubella	ICD-8: 56.0–56.9<br>ICD-10: B06.0–B06.9
Hepatitis	ICD-8: 70.0–70.9<br>ICD-10: B15.0–B15.9<br>ICD-10: B16.0–B16.9<br>ICD-10: B17.0–B19.9
Mumps	ICD-8: 72.0–72.9<br>ICD-10: B26.0–B26.9
Malaria	ICD-8: 84.0–84.9<br>ICD-10: B50.0–B54.9
Leishmaniasis	ICD-8: 85.0–85.9<br>ICD-10: B55.0–B55.9
Trypanosomiasis	ICD-8: 86.0–87.9<br>ICD-10: B56.0–B57.9
Schistosomiasis	ICD-8: 120.0–120.9<br>ICD-10: B65.0–B65.9
Other helminthiase	ICD-8: 121.0–121.9<br>ICD-8: 123.0–125.9<br>ICD-8: 127.0–129.9<br>ICD-10: B66.0–B66.9<br>ICD-10: B68.0–B75.9<br>ICD-10: B77.0–B83.9
Echinococcosis/hydatidosis	ICD-8: 122.0–122.9<br>ICD-10: B67.0–B67.9
Hookworm diseases/Ankylostomiasis	ICD-8: 126–126.9<br>ICD-10: B76.0–B76.9
Malignant neoplasm of lip, oral cavity and pharynx	ICD-8: 140.0–149.9<br>ICD-10: C00.0–C14.9
Malignant neoplasm of other digestive organs and peritoneum	ICD-8: 150.0–150.9<br>ICD-8: 155.0–159.9<br>ICD-10: C15.0–C15.9<br>ICD-10: C17.0–C17.9<br>ICD-10: C22.0–C26.9

Malignant neoplasm of stomach	ICD-8: 151.0–151.9<br>ICD-10: C16.0–C16.9
Malignant neoplasm of colon	ICD-8: 152.0–153.9<br>ICD-10: C18.0–C18.9
Malignant neoplasm of rectosigmoid junction, rectum, anus, and anal canal	ICD-8: 154.0–154.9<br>ICD-10: C19.0–C21.9
Other malignant neoplasms of respiratory and intrathoracic organs	ICD-10: C30.0–C31.9<br>ICD-10: C37.0–C39.9
Malignant neoplasm of other and unspecified respiratory organs	ICD-8: 160.0–160.9<br>ICD-8: 163.0–163.9
Malignant neoplasm of larynx	ICD-8: 161.0–161.9<br>ICD-10:C32.0–C32.9
Malignant neoplasm of trachea, bronchus and lung	ICD-8: 162.0–162.9<br>ICD-10: C33.0–C34.9
Malignant neoplasm of bone and articular cartilage	ICD-8: 170.0–170.9<br>ICD-10: C40.0–C41.9
Malignant neoplasm of skin	ICD-8: 172.0–173.9<br>ICD-10: C43.0–C43.9<br>ICD-10: C44.0–C44.9
Malignant neoplasm of other specified sites	ICD-8: 171.0–171.9<br>ICD-8: 190.0–190.9<br>ICD-8: 192.0–195.9<br>ICD-10: C45.0–C49.9<br>ICD-10: C69.0–C70.9<br>ICD-10: C72.0–C72.9
Malignant neoplasm of breast	ICD-8: I74.0–I74.9<br>ICD-10: C50.0–C50.9
Other malignant neoplasms of female genital organs	ICD-8: 181.0–181.9<br>ICD-8: 183.0–183.1<br>ICD-8: 183.0–184.9<br>ICD-10: C51.0–C52.9<br>ICD-10: C56.0–C58.9
Malignant neoplasm of *cervix uteri*	ICD-8: 180.0–180.9<br>ICD-10: C53.0–C53.9
Malignant neoplasm of other and unspecified parts of uterus	ICD-8: 182.0–182.9<br>ICD-10: C54.0–C55.9
Other malignant neoplasms of male genital organs	ICD-8: 186.0–186.9<br>ICD-10: C60.0–C60.9<br>ICD-10: C62.0–C63.9
Malignant neoplasm of prostate	ICD-8: 185.0–185.9<br>ICD-10: C61.0–C61.9
Other malignant neoplasms of urinary tract	ICD-10: C64.0–C66.9<br>ICD-10: C68.0–C68.9
Malignant neoplasm of other genitourinary	ICD-8: 187.0–187.9

organs	ICD-8: 189.0–189.9
Malignant neoplasm of bladder	ICD-8: 188.0–188.9<br>ICD-10: C67.0–C67.9
Malignant neoplasm of brain	ICD-8: 191.0–191.9<br>ICD-10: C71.0–C71.9
Malignant neoplasm of other, ill-defined, secondary, unspecified, and multiple sites	ICD-8: 197.0–199.9<br>ICD-10: C73.0–C80.<br>ICD-10: C97.0–C97.9
Hodgkin's disease	ICD-8: 201.0–201.9<br>ICD-10: C81.0–C81.9
Other malignant neoplasms of lymphoid, hematopoietic, and related tissue	ICD-8:196.0–196.9<br>ICD-8: 200.0–200.9<br>ICD-8: 202.0–203.9<br>ICD-8: 208.0–209.9<br>ICD-10: C82.0–C85.9<br>ICD-10: C88.0–C90.9<br>ICD-10: C96.0–C96.9
Leukemia	ICD-8: 204.0–207.9<br>ICD-10: C91.0–C95.9
Other *in situ* and benign neoplasms and neoplasms of uncertain and unknown behavior	ICD-8: 210.0–215.9<br>ICD-8: 217.0–217.9<br>ICD-8: 219.0–219.9<br>ICD-8: 221.0–222.9<br>ICD-8: 224.0–224.9<br>ICD-8: 226.0–228.9<br>ICD-8: 230.0–239.9<br>ICD-10: D00.0–D05.9<br>ICD-10: D07.0–D21.9<br>ICD-10: D24.0–D24.9<br>ICD-10: D26.0–D26.9<br>ICD-10: D28.0–D29.9<br>ICD-10: D31.0–D32.9<br>ICD-10: D34.0–D48.9
Carcinoma *in situ* of *cervix uteri*	ICD-8: 234.0–234.0<br>ICD-10: D06.0–D06.9
Benign neoplasm of skin	ICD-8: 216.0–216.9<br>ICD-10: D22.0–D23.9
Leiomyoma of uterus	ICD-8: 218.0–218.9<br>ICD-10: D25.0–D25.9
Benign neoplasm of ovary	ICD-8: 220.0–220.9<br>ICD-10: D27.0–D27.9
Benign neoplasm of kidney and other urinary organs	ICD-8: 223.0–223.9<br>ICD-10: D30.0–D30.9
Benign neoplasm of brain and other parts of central nervous system	ICD-8: 225.0–225.9<br>ICD-10: D33.0–D33.9
Iron deficiency anemia	ICD-8: 280.0–280.9<br>ICD-10: D50.0–D50.9

Other anemia	ICD-8: 281.0–285.9<br>ICD-10: D51.0–D64.9
Hemorrhagic conditions and other diseases of blood and blood-forming organs	ICD-8:286.0–289.9<br>ICD-10: D65.0–D77.9
Other endocrine, nutritional. and metabolic disorders	ICD-8: 251.0–258.9<br>ICD-8: 270.0–279.9<br>ICD-10: D80.0–D89.9<br>ICD-10: E15.0–E35.9<br>ICD-10: E58.0–E63.9<br>ICD-10: E65.0–E65.9<br>ICD-10: E66.0–E66.9<br>ICD-10: E67.0–E85.9<br>ICD-10: E87.0–E90.9
Other disorders of thyroid	ICD-8: 240.0–241.9<br>ICD-8: 243.0–246.9<br>ICD-10: E03.0–E04.9<br>ICD-10: E06.0–E07.9
Iodine-deficiency-related thyroid disorders	ICD-8: 242.0–242.9<br>ICD-10: E00.0–E02.9<br>ICD-10: E05.0–E05.9
Diabetes mellitus	ICD-8: 249.0–250.9<br>ICD-10: E10.0–E14.9
A-vitaminosis and other nutritional deficiency	ICD-8: 260.0–269.9<br>ICD-10: E40.0–E47.9<br>ICD-10:E50.0–E50.9<br>ICD-10: E51.0-E56.9<br>ICD-10: E64.0–E64.9
Dementia	ICD-8: 290.0–290.0<br>ICD-8: 290.0–290.9<br>ICD-10: F00.0–F03.9<br>ICD-10: G31.0–G31.0
Other mental and behavioral disorders	ICD-8: 292.0–294.9<br>ICD-8: 297.0–299.9<br>ICD-8: 305.0–309.9<br>ICD-10: F04.0–F09.9<br>ICD-10: F50.0–F69.9<br>ICD-10: F80.0–F99.9
Alcohol-, drug-abuse-related disease	ICD-8: 291.0–291.9<br>ICD-8: 303.0–304.9<br>ICD-10: F10.0–F19.9
Schizophrenia, schizotypal, and delusional disorders	ICD-8: 295.0–295.9<br>ICD-10: F20.0–F29.9
Mood (affective) disorders	ICD-8: 296.0–296.1<br>ICD-8: 296.0–296.9<br>ICD-10: F30.0–F31.9<br>ICD-10: F34.0–F39.9
Depression	ICD-8: 296.0–296.0<br>ICD-8: 296.0–296.2

	ICD-10: F32.0–F33.9
Neurotic, stress-related, and somatoform disorders	ICD-8: 300.0–302.9<br>ICD-10: F40.0–F48.9
Mental retardation	ICD-8: 310.0–315.9<br>ICD-10: F70.0–F79.9
Inflammatory diseases of the central nervous system	ICD-8: 320.0–320.9<br>ICD-8: 321.0–324.9<br>ICD-10: G00.0–G09.9
Other diseases of the nervous system	ICD-8: 330.0–333.9<br>ICD-8: 343.0–344.9<br>ICD-8: 347.0–358.9<br>ICD-10: G10.0–G13.9<br>ICD-10: G21.0–G26.9<br>ICD-10: G31.1–G32.9<br>ICD-10: G36.0–G37.9<br>ICD-10: G44.0–G44.9<br>ICD-10: G46.0–G47.9<br>ICD-10:G50.0–G73.9<br>ICD-10: G80.0–G83.9<br>ICD-10: G90.0–G99.9
Parkinson's disease	ICD-8: 342.0–342.9<br>ICD-10: G20.0–G20.9
Alzheimer's disease	ICD-8: 290.0–290.1<br>ICD-10: G30.0–G30.9
Multiple sclerosis and other demyelinating diseases	ICD-8: 340.0–341.9<br>ICD-10: G35.0–G35.9
Epilepsy	ICD-8: 345.0–345.9<br>ICD-10: G40.0–G41.9
Migraine	ICD-8: 346.0–346.9<br>ICD-10: G43.0–G43.9
Transient cerebral ischemic attacks and related syndromes	ICD-8: 435.0–435.9<br>ICD-10: G45.0–G45.9
Other inflammatory diseases of eye	ICD-8: 360.0–369.9<br>ICD-10: H00.0–H01.9<br>ICD-10: H10.0–H13.9<br>ICD-10: H15.0–H19.9
Other diseases of the eye and adnexa	ICD-8: 370.0–372.9<br>ICD-8: 377.0–379.9<br>ICD-10: H02.0–H06.9<br>ICD-10: H20.0–H22.9<br>ICD-10: H30.0–H32.9<br>ICD-10: H34.0–H36.9<br>ICD-10: H43.0–H48.9<br>ICD-10: H51.0–H59.9
Cataract and other disorders of lens	ICD-8: 374.0–374.9<br>ICD-10: H25.0–H28.9
Retinal detachments and breaks	ICD-8: 376.0–376.9

	ICD-10: H33.0–H33.9
Glaucoma	ICD-8: 375.0–375.9<br>ICD-10: H40.0–H42.9
Strabismus	ICD-8: 373.0–373.9<br>ICD-10: H49.0–H50.9
Other diseases of the ear and mastoid process	ICD-8: 380.0–380.9<br>ICD-8: 381.0–381.9<br>ICD-8: 382.0–383.9<br>ICD-8: 384.0–389.9<br>ICD-10: H60.0–H62.9<br>ICD-10: H65.0–H75.9<br>ICD-10: H80.0–H83.9<br>ICD-10: H90.0–H95.9
Acute rheumatic fever	ICD-8: 390.0–392.9<br>ICD-10: I00.0–I02.9
Chronic rheumatic heart disease	ICD-8: 393.0–392.2<br>ICD-10: I05.0–I09.9
Essential (primary) hypertension	ICD-8: 400.0–404.9<br>ICD-10: I10.0–I15.9
Angina pectoris	ICD-8: 413.0–413.9<br>ICD-10: I20.0–I20.9
Acute myocardial infarction	ICD-8: 410.0–410.9<br>ICD-10: I21.0–I22.9
Other ischemic heart diseases	ICD-8: 411.0–412.9<br>ICD-10: I23.0–I25.9
Other ischemic heart disease	ICD-8: 414.0–414.9
Pulmonary embolism	ICD-8: 450.0–450.9<br>ICD-10: I26.0–I26.9
Other heart diseases	ICD-8: 420.0–426.9<br>ICD-8: 428.0–429.9<br>ICD-10: I27.0–I43.9<br>ICD-10: I51.0–I52.9
Conduction disorders and cardiac arrhythmias	ICD-8: 427.0–427.9<br>ICD-10: I44.0–I49.9
Congestive heart failure	ICD-8: 427.0–427.0<br>ICD-10: I50.0–I50.9
Intracranial hemorrhage	ICD-8: 431.0–431.9<br>ICD-10: I60.0–I62.9
Cerebral infarction	ICD-8: 432.0–434.9<br>ICD-10: I63.0–I63.9
Other cerebrovascular diseases	ICD-8: 430.0–430.9<br>ICD-8: 436.0–436.9<br>ICD-8: 437.0–438.9<br>ICD-10: I64.0–I64.9<br>ICD-10: I65.0–I69.9

Atherosclerosis	ICD-8: 440.0–440.9<br>ICD-10: I70.0–I70.9
Other diseases of arteries, arterioles and capillaries	ICD-8: 441.0–442.9<br>ICD-8: 444.0–448.9<br>ICD-10: I71.0–I72.9<br>ICD-10: I74.0–I74.9<br>ICD-10:I77.0–I79.9
Other peripheral vascular diseases	ICD-8: 443.0–443.9<br>ICD-10: I73.0–I73.9
Phlebitis, thrombophlebitis, venous embolism and thrombosis	ICD-8: 451.0–453.9<br>ICD-10: I80.0–I82.9
Varicose veins of lower extremities	ICD-8: 454.0–454.9<br>ICD-10: I83.0–I83.9
Hemorrhoids	ICD-8: 455.0–455.9<br>ICD-10: I84.0–I84.9
Other diseases of the circulatory system	ICD-8: 456.0–458.9<br>ICD-10: I85.0–I99.9
Other acute upper respiratory infections	ICD-8: 460–461.9<br>ICD-8: 464.0–465.9<br>ICD-10: J00.0–J01.9<br>ICD-10: J04.0–J04.9<br>ICD-10: J05.0–J06.9
Acute pharyngitis and acute tonsillitis	ICD-8:34.0–34.0<br>ICD-8: 462.0–463.9<br>ICD-10: J02.0–J03.9
Influenza	ICD-8: 470.0–474.9<br>ICD-10: J10.0–J11.9
Pneumonia	ICD-8: 480.0–480.9<br>ICD-8: 481.0–481.9<br>ICD-8: 482.0–483.9<br>ICD-8: 484.0–486.9<br>ICD-10: J12.0–J18.9
Acute bronchitis and acute bronchiolitis	ICD-8: 466.0–466.9<br>ICD-10: J20.0–J21.9
Other diseases of the respiratory system	ICD-8: 510.0–514.9<br>ICD-8: 517.0–517.9<br>ICD-8: 519.0–519.9<br>ICD-10: J22.0–J22.9<br>ICD-10: J66.0–J99.9
Other diseases of upper respiratory tract	ICD-8: 501.0–502.9<br>ICD-8: 504.0–504.9<br>ICD-8: 505.0–508.9<br>ICD-10: J30.0–J31.9<br>ICD-10: J33.0–J34.9<br>ICD-10: J36.0–J39.9
Chronic sinusitis	ICD-8: 503.0–503.9<br>ICD-10: J32.0–J32.9

Chronic disease of tonsils and adenoids	ICD-8: 500.0–500.9<br>ICD-10: J35.0–J35.9
Bronchitis, emphysema and other chronic pulmonary diseases	ICD-8: 490.0–493.9<br>ICD-10: J40.0–J44.9<br>ICD-10: J45.0–J46.9
Bronchiectasis	ICD-8: 518.0–518.9<br>ICD-10: J47.0–J47.9
Pneumoconioses and related diseases	ICD-8: 515.0–516.9<br>ICD-10: J60.0–J65.9
Other diseases of the teeth, oral cavity, salivary glands and jaws	ICD-8: 520.0–529.9<br>ICD-10: K00.0–K14.9
Other diseases of esophagus, stomach and duodenum	ICD-8: 530.0–530.9<br>iCD-8: 536.0–537.9<br>ICD-10: K20.0–K23.9<br>ICD-10: K28.0–K28.9<br>ICD-10: K30.0–K31.9
Gastric and duodenal ulcer	ICD-8: 531.0–534.9<br>ICD-10: K25.0–K27.9
Gastritis and duodenitis	ICD-8: 535.0–535.9<br>ICD-10: K29.0–K29.9
Diseases of appendix	ICD-8: 540.0–543.9<br>ICD-10: K35.0–K38.9
Hernia	ICD-8: 550.0–553.9<br>ICD-10: K40.0–K46.9
Crohn’s disease and ulcerative colitis	ICD-8: 563.0–563.9<br>ICD-10: K50.0–K51.9
Other diseases of the digestive system	ICD-8: 561.0–562.9<br>ICD-8: 564.0–569.9<br>ICD-10: K52.0–K55.9<br>ICD-10: K57.0–K67.9<br>ICD-10: K82.0–K83.9<br>ICD-10: K87.0–K93.9
Paralytic ileus and intestinal obstruction without hernia	ICD-8: 560.0–560.9<br>ICD-10: K56.0–K56.9
Other diseases of liver and gallbladder	ICD-8: 570.0–573.9<br>ICD-8: 576.0–576.9<br>ICD-10: K70.0–K77.9
Cholelithiasis and cholecystitis	ICD-8: 574.0–575.9<br>ICD-10: K80.0–K81.9
Acute pancreatitis and other diseases of the pancreas	ICD-8:577.0–577.9<br>ICD-10: K85.0–K86.9
Infections of the skin and subcutaneous tissue	ICD-8: 680.0–686.9<br>ICD-10: L00.0–L08.9
Other diseases of the skin and subcutaneous tissue	ICD-8:690.0–698.9<br>ICD-8: 700.0–709.9

	ICD-10: L10.0–L99.9
Other disorders of joints	ICD-8: 724.0–724.9<br>ICD-8: 726.0–727.9<br>ICD-8:729.0–729.9<br>ICD-8:737.0–737.9<br>ICD-10: M00.0–M03.9<br>ICD-10: M22.0–M25.9
Rheumatoid arthritis and other inflammatory polyarthropathies	ICD-8: 712.0–712.9<br>ICD-8: 716.0–716.9<br>ICD-10: M05.0–M14.9
Osteoarthritis and allied conditions	ICD-8: 710.0–711.9<br>ICD-8: 713.0–715.9<br>ICD-10: M15.0–M19.9<br>ICD-10: M47.0–M47.9<br>ICD-10: M48.3–M48.3
Acquired deformities of limbs	ICD-8: 736.0–736.9<br>ICD-10: M20.0–M21.9
Other diseases of the musculoskeletal system and connective tissue	ICD-8: 730.0–730.9<br>ICD-8: 733.0–734.9<br>ICD-8: 738.0–738.9<br>ICD-10: M30.0–M36.9<br>ICD-10: M87.0–M90.9<br>ICD-10: M94.0–M99.9
Other dorsopathies	ICD-8: 735.0–735.9<br>ICD-10: M40.0–M41.9<br>ICD-10: M43.0–M43.5<br>ICD-10: M43.7-M46.9<br>ICD-10: M48.0–M48.2<br>ICD-10: M48.4–M49.9<br>ICD-10: M53.0–M53.9
Osteochondrosis	ICD-8: 722.0–722.9<br>ICD-10: M42.0–M42.9<br>ICD-10: M91.0–M93.9
Rheumatism	ICD-8: 717.0–718.9<br>ICD-10: M43.6–M43.6<br>ICD-10: M79.0–M79.1
Cervical and other intervertebral disc disorders	ICD-8: 725.0–725.9<br>ICD-8: 728.0–728.9<br>ICD-10: M50.0–M51.9<br>ICD-10: M54.0–M54.9
Myositis	ICD-8: 732.0–732.9<br>ICD-10: M60.0–M60.9
Soft tissue disorders	ICD-8: 731.0–731.9<br>ICD-10: M61.0–M78.9<br>ICD-10: M79.2–M79.5<br>ICD-10: M79.7–M79.9
Osteoporosis with and without fracture	ICD-8: 723.0–723.0<br>ICD-10: M80.0–M81.9

Other diseases of bone	ICD-8: 721.0–721.9<br>ICD-8: 723.0–723.9<br>ICD-10: M82.0–M85.9
Osteomyelitis and periostitis	ICD-8: 720.0–720.9<br>ICD-10: M86.0–M86.9
Nephritis and nephrosis	ICD-8: 580.0–584.9<br>ICD-10: N00.0–N08.9
Infections of kidney	ICD-8: 590.0–590.9<br>ICD-10: N10.0–N16.9
Other diseases of the urinary system	ICD-8: 591.0–591.9<br>ICD-8: 593.0–593.9<br>ICD-8:596.0–599.9<br>ICD-10: N17.0–N19.9<br>ICD-10: N25.0–N29.9<br>ICD-10: N31.0–N39.9
Urolithiasis/Calculus of urinary system	ICD-8: 592.0–592.9<br>ICD-8: 594.0–594.9<br>ICD-10: N20.0–N23.9
Cystitis	ICD-8: 595.0–595.9<br>ICD-10: N30.0–N30.9
Hyperplasia of prostate	ICD-8: 600.0–600.9<br>ICD-10: N40.0–N40.9
Other diseases of male genital organs	ICD-8: 601.0–602.9<br>ICD-8: 604.0–604.9<br>ICD-8: 606.0–607.9<br>ICD-10: N41.0–N42.9<br>ICD-10: N44.0–N46.9<br>ICD-10: N48.0–N51.9
Hydrocele and spermatocele	ICD-8: 603.0–603.9<br>ICD-10: N43.0–N43.9
Redundant prepuce, phimosis and paraphimosis	ICD-8: 605.0–605.9<br>ICD-10: N47.0–N47.9
Disorders of breast	ICD-8: 610.0–611.9<br>ICD-10: N60.0–N64.9
Salpingitis and oophoritis	ICD-8: 612.0–614.9<br>ICD-10: N70.0–N70.9
Other inflammatory diseases of female pelvic organs	ICD-8: 622.0–622.9<br>ICD-10:N71.0–N71.9<br>ICD-10: N73.0–N77.9
Inflammatory disease of cervix uteri	ICD-8: 620.0–620.9<br>ICD-10: N72.0–N72.9
Endometriosis	ICD-8: 625.0–625.3<br>ICD-10: N80.0–N80.9
Female genital prolapse	ICD-8: 623.0–623.9<br>ICD-10: N81.0–N81.9

Other disorders of genitourinary tract	ICD-8:621.0–621.9<br>ICD-8: 624.0–625.9<br>ICD-8: 627.0–627.9<br>ICD-8: 629.0–629.9<br>ICD-10: N82.0–N82.9<br>ICD-10: N84.0–N90.9<br>ICD-10: N93.0–N96.9<br>ICD-10: N98.0–N99.9
Other diseases of ovary, fallopian tube and parametrium	ICD-8: 615.0–616.9<br>ICD-10: N83.0–N83.9
Disorders of menstruation	ICD-8: 626.0–626.9<br>ICD-10: N91.0–N92.9
Female infertility	ICD-8: 628.0–628.9<br>ICD-10: N97.0–N97.9
Ectopic pregnancy	ICD-8: 631.0–631.9<br>ICD-10: O00.0–O00.9
Pregnancies with abortive outcome	ICD-8: 640.0–645.9<br>ICD-10: O01.0–O08.9
Other complications of pregnancy or delivery	ICD-8: 630.0–639.9<br>ICD-8: 651.0–666.9<br>ICD-8: 670.0–678.9<br>ICD-10: O10.0–O16.9<br>ICD-10: O20.0–O48.9<br>ICD-10: O60.0–O75.9<br>ICD-10: O81.0–O99.9
Delivery without mention of complication	ICD-8: 650.0–650.9<br>ICD-10: O80.0–O80.9
Conditions originating in the perinatal period	ICD-8: 760.0–773.9<br>ICD-8: 776.0–779.9<br>ICD-10: P00.0–P54.9<br>ICD-10: P56.0–P96.9
Hemolytic disease of fetus and newborn	ICD-8: 774.0–775.9<br>ICD-10: P55.0–P55.9
Spina bifida and congenital hydrocephalus	ICD-8: 741.0–742.9<br>ICD-10: Q05.0–Q05.9
Congenital malformations of the circulatory system	ICD-8: 746.0–747.9<br>ICD-10: Q20.0–Q28.9
Cleft lip and cleft palate	ICD-8: 749.0–749.9<br>ICD-10: Q35.0–Q37.9
Other congenital malformations of the digestive system	ICD-8: 750.0–750.0<br>ICD-8: 750.0–750.9<br>ICD-8: 751.0–751.9<br>ICD-10: Q38.0–Q40.9<br>ICD-10: Q42.0–Q45.9
Absence, atresia and stenosis of small intestine	ICD-8: 750.0–750.1<br>ICD-10: Q41.0–Q41.9

Other malformations of the genitourinary system	ICD-8: 752.2–753.9<br>ICD-10: Q50.0–Q52.9<br>ICD-10: Q54.0–Q64.9
Undescended testicle	ICD-8: 752.0–752.1<br>ICD-10: Q53.0–Q53.9
Congenital deformities of hip	ICD-8: 755.0–755.6<br>ICD-10: Q65.0–Q65.9
Congenital deformities of feet	ICD-8: 754.0–754.9<br>ICD-10: Q66.0–Q66.9
Other and unspecified congenital anomalies	ICD-8: 740.0–740.9<br>ICD-8: 743.0–745.9<br>ICD-8: 748.0–748.9<br>ICD-8: 757.0–759.9<br>ICD-10: Q00–Q04.9<br>ICD-10:Q06.0–Q07.9<br>ICD-10: Q10–Q18.9<br>ICD-10: Q30.0–Q34.9<br>ICD-10: Q80.0–Q99.9
Other congenital malformations and deformations of the musculoskeletal system	ICD-8: 755.0–755.9<br>ICD-8: 755.0–756.9<br>ICD-8: 756.0–756.9<br>ICD-10: Q67.0–Q79.9
Abdominal and pelvic pain	ICD-8: 785.0–785.5<br>ICD-10: R10.0–R10.9
Senility	ICD-8: 794.0–794.9<br>ICD-10: R54.0–R54.9
‘Symptoms, signs and abnormal clinical and laboratory findings not otherwise specified’	ICD-10: Rxx
‘Injury, poisoning and certain other consequences of external causes’	ICD-10: DSxx and DTxx
‘Observation for or follow-up after treatment for cancer’	ICD-10: Z031 and DZ08
‘Contact with health services due to solid organ or bone marrow transplantation, and other factors influencing health status and contact with health services’	Remaining codes

Abbreviations: ICD = International Classification of Diseases; NOMESCO = Nordico Medico-Statistical Committee

[a]Primary diagnoses among patients with secondary herpes zoster diagnosis were categorized according to a list of 203 morbidity groups based on the ICD-8 and ICD-10 World Health Organization morbidity tables. This code list was adapted from a previously published study (Ording AG, Garne JP, Nyström PMW, Cronin-Fenton D, Tarp M, Sørensen HT, *et al*. Hospital recorded morbidity and breast cancer incidence: a nationwide population-based case-control study. PLoS One. 2012;7(10):e47329). Additional codes falling outside this categorization comprised general and unspecific for hospital contact and were grouped by us in four additional categories (‘Symptoms, signs and abnormal clinical and laboratory findings not otherwise specified’, ‘Injury, poisoning and certain other consequences of external causes’, ‘Observation for or follow-up after treatment for cancer’, and ‘Contact with health services due to solid organ or bone marrow transplantation, and other factors influencing health status and contact with health services’).

Table S2. Reasons for hospital contact among patients with a secondary herpes zoster diagnosis, Denmark, 1994–2012

**Primary diagnosis**[a]	**No.**	**%**
Other factors influencing health status and contact with health services	309	8.1%
Symptoms, signs and abnormal clinical and laboratory findings not otherwise specified	307	8.0%
Pneumonia	227	5.9%
Bronchitis, emphysema and other obstructive pulmonary diseases	184	4.8%
Other endocrine, nutritional and metabolic disorders	165	4.3%
Other viral diseases	151	3.9%
Injury, poisoning and certain other consequences of external causes	144	3.8%
Other malignant neoplasm of lymphoid, hematopoietic and related tissue	135	3.5%
Leukemia	115	3.0%
Other diseases of the digestive system	108	2.8%
Other disease of the nervous system	107	2.8%
Other diseases of the skin and subcutaneous tissue	101	2.6%
Septicemia	75	2.0%
Other cerebrovascular disease	69	1.8%
Cystitis	64	1.7%
Other bacterial disease	63	1.6%
Other diseases of the urinary system	62	1.6%
Conduction disorders and cardiac arrhythmia	57	1.5%
Other inflammatory disease of the eye	52	1.4%
Diabetes mellitus	52	1.4%
Congestive heart failure	48	1.3%
Angina pectoris	43	1.1%
Other diseases of the eye and adnexa	43	1.1%
Other anemia	43	1.1%
Other diseases of the musculoskeletal system and connective tissue	43	1.1%
Cervical and other intervertebral disk	43	1.1%
Other disease of the respiratory system	42	1.1%
Other diseases of the ear and mastoid process	33	0.9%
Cerebral infarction	33	0.9%
Infections of the skin and subcutaneous and subcutaneous tissue	31	0.8%
Essential (primary) hypertension	28	0.7%
Diarrhea and gastro-enteritis of presumed infectious origin	28	0.7%
Soft tissue disorders	28	0.7%
Contact with health services due to transplanted organ	27	0.7%
Acute myocardial infarction	26	0.7%
Other infectious and parasitic disease	23	0.6%
Observation for or follow-up after treatment for cancer	22	0.6%
Gastric and duodenal ulcer	22	0.6%
Dementia	20	0.5%
Cataract and other disorders of lens	20	0.5%
Other diseases of liver and gallbladder	20	0.5%
Infections of the skin and subcutaneous and subcutaneous tissue	20	0.5%
Osteoarthritis and allied conditions	20	0.5%
Osteoporosis w/o fracture	20	0.5%
Malignant neoplasm of trachea, bronchus and lung	18	0.5%

Other ischemic heart disease	17	0.4%
Other in situ and benign neoplasms and neoplasms of uncertain and unknown behavior	17	0.4%
Diseases of appendix	17	0.4%
Malignant neoplasm of breast	15	0.4%
Transient cerebral ischemic attacks and related syndromes	15	0.4%
Other diseases of the teeth, oral cavity, salivary glands and jaws	15	0.4%
Inflammatory disease of the central nervous system	14	0.4%
Malignant neoplasm of other, ill-defined, secondary and multiple sites	14	0.4%
Infections of kidney	13	0.3%
Gastritis and duodenitis	12	0.3%
Crohn's disease and ulcerative colitis	12	0.3%
Glaucoma	11	0.3%
Phlebitis	11	0.3%
Malignant neoplasm of prostate	11	0.3%
Other mental and behavioral disorders	11	0.3%
Viral encephalitis	11	0.3%
Urolithiasis/calculus of urinary system	11	0.3%
Other complications of pregnancy or delivery	11	0.3%
Other diseases of esophagus, stomach and duodenum	11	0.3%
Other intestinal infectious disease	10	0.3%
Hemorrhagic conditions and other diseases of blood and blood-forming organs	10	0.3%
Malignant neoplasm of brain	10	0.3%
Hodgkin's disease	10	0.3%
Epilepsy	10	0.3%
Pulmonary embolism	10	0.3%

[a] Only diagnoses with 10 or more persons recorded are shown.

Table S3. Characteristics of 13,663 patients with a first-time hospital-based diagnosis of HZ, Denmark, 1994–2012, by diagnosis types

	Type of diagnosis				Type of hospital contact					
	Primary diagnosis (n=9,835)		Secondary diagnosis (n=3,828)		Inpatient (n=8,228)		Outpatient (n=3,405)		Emergency (n=2,030)	
	No.	%	No.	%	No.	%	No.	%	No.	%
**Type of hospital contact**										
Outpatient	2,874	29.2%	531	13.9%	–	–				
Inpatient	5,049	51.3%	3,179	83.0%			–	–		
Emergency	1,912	19.4%	118	3.1%	–	–	–	–		
**Type of diagnosis**										
Primary			–	–	5,049	61.4%	2,874	84.4%	1,912	94.2%
Secondary	–	–			3,179	38.6%	531	15.6%	118	5.8%
**Diagnosis code (ICD-10 code)**										
HZ meningo-encephalitis (B02.0–1)	264	2.7%	73	1.9%	290	3.5%	42	1.2%	5	0.2%
HZ encephalitis (B02.0)	199	2.0%	64	1.7%	222	2.7%	36	1.1%	5	0.2%
HZ meningitis (B02.1)	65	0.7%	9	0.2%	68	0.8%	6	0.2%	0	0.0%
HZ with other nervous system involvement (B02.2)	822	8.4%	239	6.2%	631	7.7%	322	9.5%	108	5.3%
HZ ophthalmicus (B02.3)	1,449	14.7%	253	6.6%	462	5.6%	951	27.9%	289	14.2%
Disseminated HZ (B02.7)	200	2.0%	73	1.9%	213	2.6%	42	1.2%	18	0.9%
HZ with other complication (B02.8)	480	4.9%	179	4.7%	375	4.6%	204	6.0%	80	3.9%
HZ without complication (B02.9)	6,620	67.3%	3,011	78.7%	6,257	76.0%	1,844	54.2%	1,530	75.4%
**Age (years)**										
0–9	393	4.0%	99	2.6%	306	3.7%	126	3.7%	60	3.0%
10–19	294	3.0%	74	1.9%	202	2.5%	111	3.3%	55	2.7%
20–29	525	5.3%	98	2.6%	251	3.1%	176	5.2%	196	9.7%
30–39	602	6.1%	170	4.4%	305	3.7%	264	7.8%	203	10.0%
40–49	696	7.1%	189	4.9%	404	4.9%	295	8.7%	186	9.2%
50–59	1,297	13.2%	353	9.2%	775	9.4%	520	15.3%	355	17.5%
60–69	1,712	17.4%	645	16.8%	1,361	16.5%	636	18.7%	360	17.7%
70–79	2,109	21.4%	985	25.7%	2,092	25.4%	660	19.4%	342	16.8%
80–89	1,862	18.9%	997	26.0%	2,089	25.4%	541	15.9%	229	11.3%
≥90	345	3.5%	218	5.7%	443	5.4%	76	2.2%	44	2.2%
**Sex**										
Women	5,614	57.1%	2,209	57.7%	4,812	58.5%	1,940	57.0%	1,071	52.8%
Men	4,221	42.9%	1,619	42.3%	3,416	41.5%	1,465	43.0%	959	47.2%
**Any of the comorbidities identified**	5,306	54.0%	2,761	72.1%	5,637	68.5%	1,637	48.1%	793	39.1%
**Charlson Comorbidity Index level**										
None	4,832	49.1%	1,226	32.0%	2,849	34.6%	1,919	56.4%	1,290	63.5%
Moderate	1,647	16.7%	717	18.7%	1,515	18.4%	538	15.8%	311	15.3%
Severe	1,552	15.8%	822	21.5%	1,727	21.0%	439	12.9%	208	10.2%
Very Severe	1,804	18.3%	1,063	27.8%	2,137	26.0%	509	14.9%	221	10.9%

Myocardial infarction	601	6.1%	320	8.4%	656	8.0%	171	5.0%	94	4.6%
Congestive heart failure	626	6.4%	424	11.1%	804	9.8%	166	4.9%	80	3.9%
Peripheral vascular disease	529	5.4%	274	7.2%	573	7.0%	164	4.8%	66	3.3%
Cerebrovascular disease	910	9.3%	536	14.0%	1,045	12.7%	272	8.0%	129	6.4%
Dementia	156	1.6%	100	2.6%	180	2.2%	40	1.2%	36	1.8%
Chronic pulmonary disease	1,196	12.2%	715	18.7%	1,351	16.4%	356	10.5%	204	10.0%
Connective tissue disease	681	6.9%	302	7.9%	695	8.4%	206	6.0%	82	4.0%
Ulcer disease	545	5.5%	303	7.9%	602	7.3%	161	4.7%	85	4.2%
Mild liver disease	108	1.1%	63	1.6%	88	1.1%	58	1.7%	25	1.2%
Diabetes without end organ damage	598	6.1%	320	8.4%	655	8.0%	177	5.2%	86	4.2%
Hemiplegia	39	0.4%	18	0.5%	42	0.5%	12	0.4%	3	0.1%
Moderate to severe renal disease	439	4.5%	204	5.3%	461	5.6%	140	4.1%	42	2.1%
Diabetes with end organ damage	268	2.7%	151	3.9%	312	3.8%	77	2.3%	30	1.5%
Non–metastatic solid tumor	1,156	11.8%	599	15.6%	1,199	14.6%	386	11.3%	170	8.4%
Leukemia	309	3.1%	180	4.7%	429	5.2%	41	1.2%	19	0.9%
Lymphoma	550	5.6%	284	7.4%	715	8.7%	85	2.5%	34	1.7%
Moderate to severe liver disease	37	0.4%	25	0.7%	40	0.5%	17	0.5%	5	0.2%
Metastatic solid tumor	204	2.1%	127	3.3%	236	2.9%	69	2.0%	26	1.3%
Acquired immune deficiency syndrome	70	0.7%	83	2.2%	113	1.4%	22	0.6%	18	0.9%
**Comorbidities associated with immunosuppression**										
Stem cell or bone marrow transplantation	207	2.1%	66	1.7%	245	3.0%	19	0.6%	9	0.4%
Solid organ transplantation	113	1.1%	39	1.0%	117	1.4%	27	0.8%	8	0.4%
Human immunodeficiency virus infection	94	1.0%	116	3.0%	162	2.0%	29	0.9%	19	0.9%
Primary immunodeficiency	41	0.4%	20	0.5%	48	0.6%	10	0.3%	3	0.1%
Any cancer	1,851	18.8%	970	25.3%	2,128	25.9%	483	14.2%	210	10.3%
Any autoimmune disease	1,524	15.5%	769	20.1%	1,557	18.9%	532	15.6%	204	10.0%
Hematological	46	0.5%	27	0.7%	59	0.7%	12	0.4%	2	0.1%
Endocrine	404	4.1%	210	5.5%	436	5.3%	127	3.7%	51	2.5%
Central nervous system	44	0.4%	23	0.6%	43	0.5%	18	0.5%	6	0.3%
Gastrointestinal	189	1.9%	85	2.2%	172	2.1%	67	2.0%	35	1.7%
Skin	202	2.1%	113	3.0%	182	2.2%	94	2.8%	39	1.9%
Connective tissue disease	733	7.5%	347	9.1%	771	9.4%	229	6.7%	80	3.9%
Pulmonary	23	0.2%	8	0.2%	24	0.3%	7	0.2%	0	0.0%
Ocular	60	0.6%	34	0.9%	40	0.5%	49	1.4%	5	0.2%

Abbreviations: HZ, herpes zoster

Table S4. SMRs among patients with a first-time hospital-based diagnosis of HZ, Denmark, 1994–2012, by diagnosis types

	Observed	Expected	SMR (95% CI)[a]
**Overall**	6,059	3,414	1.8 (1.7–1.8)
**Type of hospital contact**			
Emergency room	562	447	1.3 (1.2–1.4)
Outpatient clinic	1,090	753	1.4 (1.4–1.5)
Inpatient	4,407	2,214	2.0 (1.9–2.1)
**Type of diagnosis**			
Primary diagnosis	3,854	2,493	1.5 (1.5–1.6)
Secondary diagnosis	2,205	920	2.4 (2.3–2.5)
**Severity of HZ**			
Uncomplicated	4,429	2,382	1.9 (1.8–1.9)
Complicated	1,630	1,031	1.6 (1.5–1.7)
**Extent of HZ**			
Localized	5,051	2,846	1.8 (1.7–1.8)
Disseminated	1,008	568	1.8 (1.7–1.9)
**Diagnosis code (ICD-10 code)**			
HZ meningo-encephalitis (B02.0–1)	112	60	1.8 (1.5–2.2)
HZ encephalitis (B02.0)	103	54	1.9 (1.6–2.3)
HZ meningitis (B02.1)	9	6.4	1.4 (0.7–2.7)
HZ with other nervous system involvement (B02.2)	454	298	1.5 (1.4–1.7)
HZ ophthalmicus (B02.3)	622	464	1.3 (1.2–1.5)
Disseminated HZ (B02.7)	150	40	3.7 (3.2–4.4)
HZ with other complication (B02.8)	292	170	1.7 (1.5–1.9)
HZ without complication (B02.9)	4,429	2,382	1.9 (1.8–1.9)

Abbreviations: CI, confidence interval; HZ, herpes zoster; SMR, Standardized mortality ratios
[a] Computed using indirect standardization with the Danish general population as reference.

Figure S1. Rate of first-time hospital-based diagnoses of herpes zoster among men and women, Denmark, 1994–2012, directly standardized to the age-distribution in the 2000 Danish Census

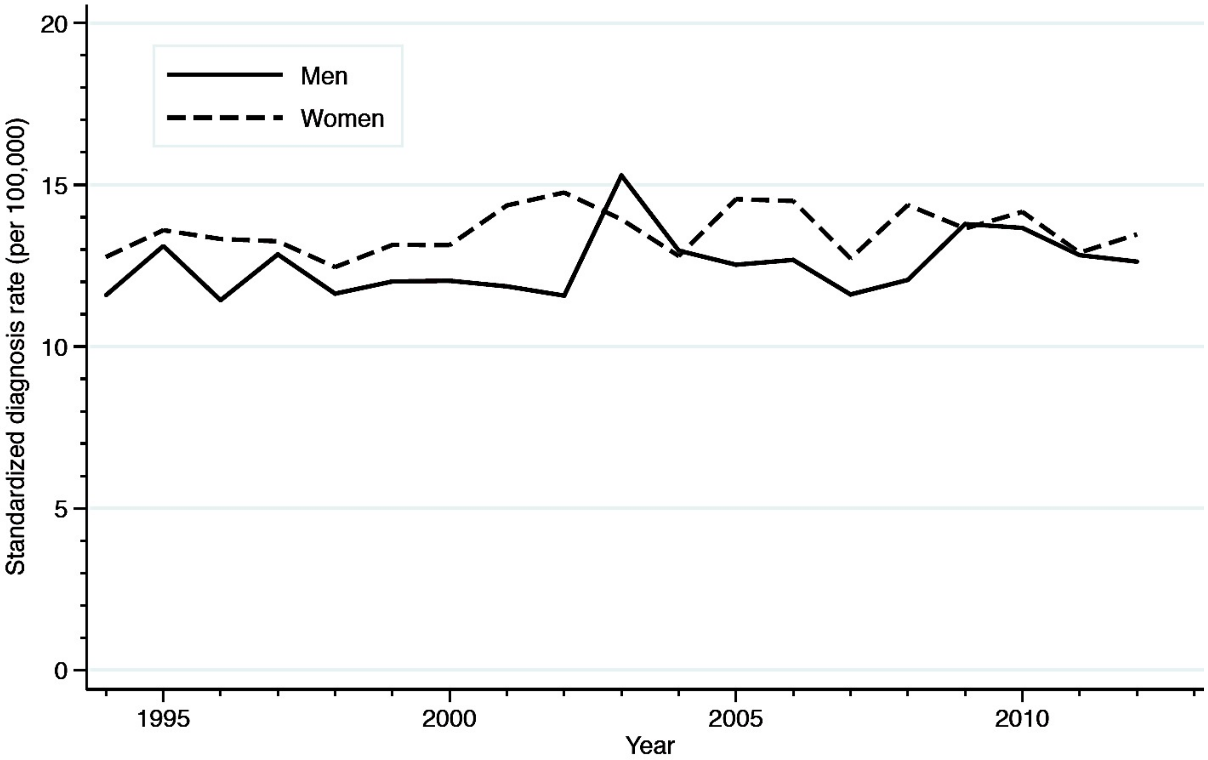

Figure S2. Age-specific rates of first-time hospital-based diagnoses of herpes zoster, Denmark, 1994–2012

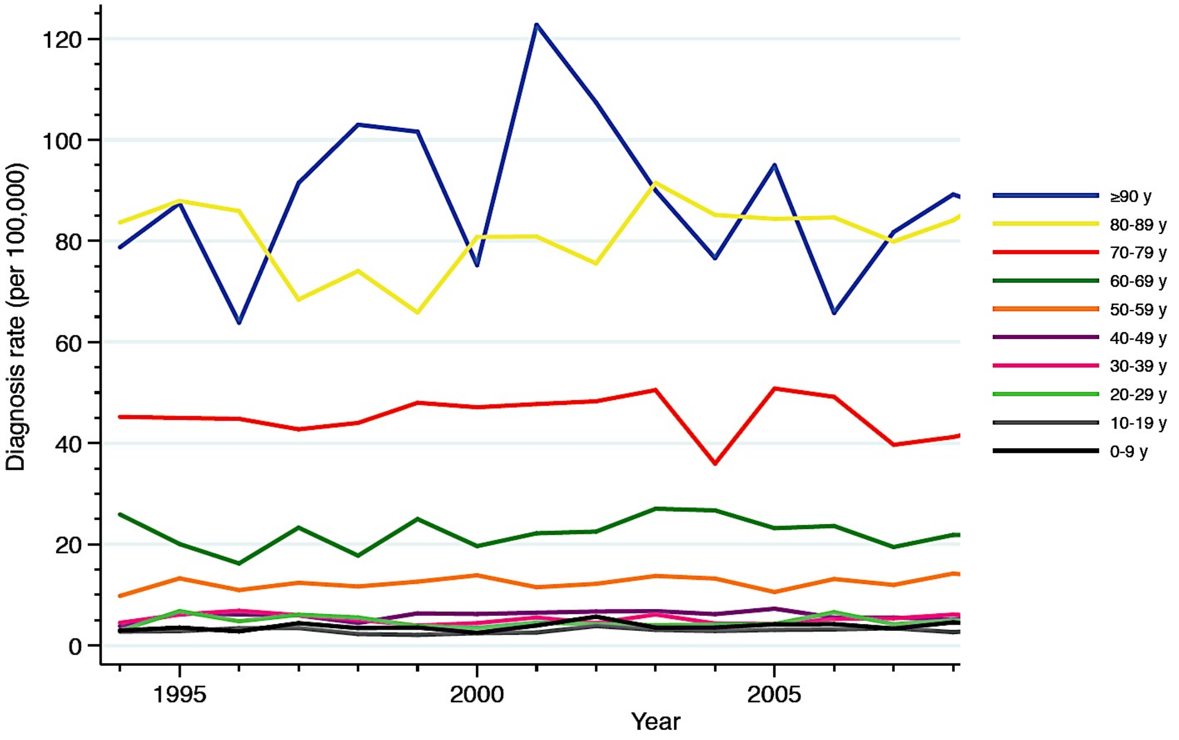

Figure S3. Age-specific diagnosis rates of herpes zoster reported for European countries

a) Primary inpatient diagnoses of herpes zoster

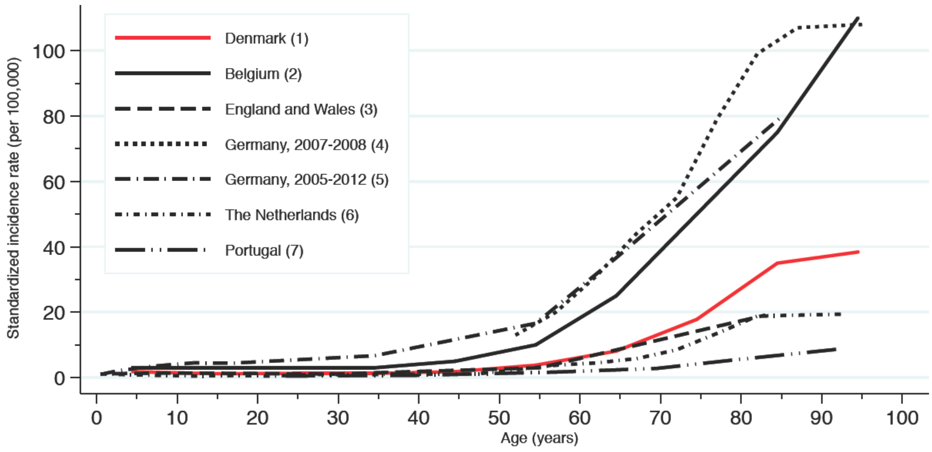

b) Primary and secondary inpatient diagnoses of herpes zoster

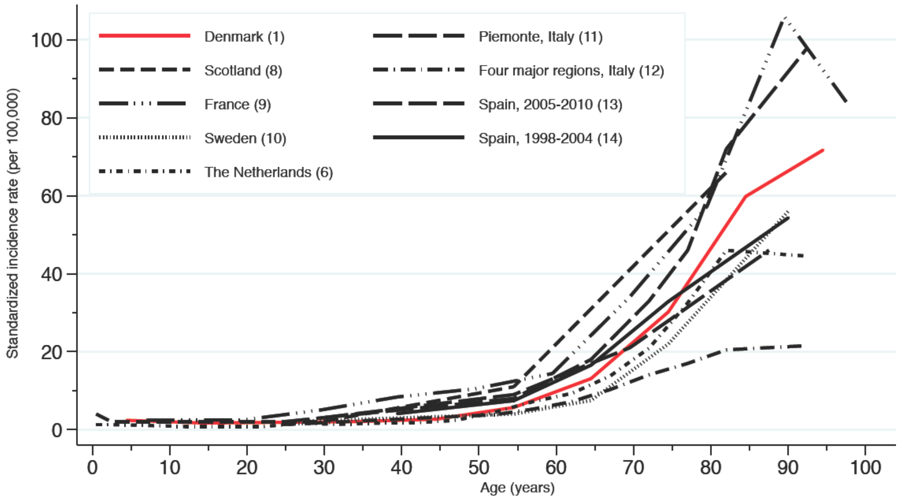

(1) Present study
(2) Bilcke J, Ogunjimi B, Marais C, de Smet F, Callens M, Callaert K, et al. The health and economic burden of chickenpox and herpes zoster in Belgium. Epidemiol Infect. 2012;140(11):2096–109.
(3) Brisson M, Edmunds WJ. Epidemiology of Varicella-Zoster Virus in England and Wales. J Med Virol. 2003;70 Suppl 1:S9–14.
(4) Ultsch B, Siedler A, Rieck T, Reinhold T, Krause G, Wichmann O. Herpes zoster in Germany: quantifying the burden of disease. BMC Infect Dis. 2011;11:173.
(5) Siedler A, Dettmann M. Hospitalization with varicella and shingles before and after introduction of childhood varicella vaccination in Germany. Hum Vaccin Immunother. 2014;10(12):3594–600.
(6) de Melker H, Berbers G, Hahné S, Rümke H, van den Hof S, de Wit A, et al. The epidemiology of varicella and herpes zoster in The Netherlands: implications for varicella zoster virus vaccination. Vaccine. 2006;24(18):3946–52.
(7) Mesquita M, Froes F. Hospital admissions for herpes zoster in Portugal between 2000 and 2010. Acta Med Port. 2013 Sep;26(5):531–6.
(8) Scott FT, Johnson RW, Leedham-Green M, Davies E, Edmunds WJ, Breuer J. The burden of Herpes Zoster: a prospective population based study. Vaccine. 2006 Feb 27;24(9):1308–14.
(9) Gonzalez Chiappe S, Sarazin M, Turbelin C, Lasserre A, Pelat C, Bonmarin I, et al. Herpes zoster: Burden of disease in France. Vaccine. 2010 Nov 23;28(50):7933–8.
(10) Studahl M, Petzold M, Cassel T. Disease burden of herpes zoster in Sweden--predominance in the elderly and in women - a register based study. BMC Infect Dis. 2013;13:586.
(11) Di Legami V, Gianino MM, Ciofi degli Atti M, Massari M, Migliardi A, Tomba GS, et al. Epidemiology and costs of herpes zoster: background data to estimate the impact of vaccination. Vaccine. 2007 Oct 23;25(43):7598–604.
(12) Gialloreti LE, Merito M, Pezzotti P, Naldi L, Gatti A, Beillat M, et al. Epidemiology and economic burden of herpes zoster and post-herpetic neuralgia in Italy: a retrospective, population-based study. BMC Infect Dis. 2010;10:230.
(13) Gil-Prieto R, Walter S, Gonzalez-Escalada A, Garcia-Garcia L, Marín-García P, Gil-de-Miguel A. Different vaccination strategies in Spain and its impact on severe varicella and zoster. Vaccine. 2014 Jan 3;32(2):277–83.
(14) Gil A, Gil R, Alvaro A, San Martín M, González A. Burden of herpes zoster requiring hospitalization in Spain during a seven-year period (1998-2004). BMC Infect Dis. 2009;9:55.
